# Targeting the core program of metastasis with a novel drug combination

**DOI:** 10.1002/cam4.7291

**Published:** 2024-06-03

**Authors:** Gulimirerouzi Fnu, Georg F. Weber

**Affiliations:** ^1^ James L. Winkle College of Pharmacy University of Cincinnati Academic Health Center Cincinnati Ohio USA

**Keywords:** extracellular matrix, gene expression, ion homeostasis, metabolism, metastasis, vascularization

## Abstract

**Background:**

We previously reported that metastases are generally characterized by a core program of gene expression that activates tissue remodeling/vascularization, alters ion homeostasis, induces the oxidative metabolism, and silences extracellular matrix interactions. This core program distinguishes metastases from their originating primary tumors as well as from their destination host tissues. Therefore, the gene products involved are potential targets for anti‐metastasis drug treatment.

**Methods:**

Because the silencing of extracellular matrix interactions predisposes to anoiks in the absence of active survival mechanisms, we tested inhibitors against the other three components.

**Results:**

Individually, the low‐specificity VEGFR blocker pazopanib (in vivo combined with marimastat), the antioxidant dimethyl sulfoxide (or the substitute atovaquone, which is approved for internal administration), and the ionic modulators bumetanide and tetrathiomolybdate inhibited soft agar colony formation by breast and pancreatic cancer cell lines. The individual candidate agents have a record of use in humans (with limited efficacy when administered individually) and are available for repurposing. In combination, the effects of these drugs were additive or synergistic. In two mouse models of cancer (utilizing 4T1 cells or B16‐F10 cells), the combination treatment with these medications, applied immediately (to prevent metastasis formation) or after a delay (to suppress established metastases), dramatically reduced the occurrence of disseminated foci.

**Conclusions:**

The combination of tissue remodeling inhibitors, suppressors of the oxidative metabolism, and ion homeostasis modulators has very strong promise for the treatment of metastases by multiple cancers.

## 1 INTRODUCTION

Even though the chemotherapy of primary tumors has been increasingly successful, the drug treatment of metastases has been compromised by low success rates. A likely reason lies in the clinical proclivity to treat the disseminated growths with the same agents that have been devised to counteract the primary tumors. Metastases, however, evolve their gene expression patterns to differentiate them from their originating tumors as well as from the implantation organ.^1^ There are site‐specific changes,^2^ but they would be difficult to control with chemotherapy, because each site of dissemination would require a separate set of drugs. The core signature of metastases in general offers the possibility for intervention. Drug cocktails, which impact the phenotypic characteristics that are uniquely imparted by the gene expression programs of all metastases, may be most suitable to eradicate disseminated cancers.

Since the mid‐1980s (with the discovery of individual genes that direct or suppress metastasis), it has become increasingly apparent that the phenomenon of cancer dissemination can be mediated by gene expression programs within the transformed cells.^3^, ^4^ Metastatic potential is acquired by these neoplastic cells through the aberrant expression or splicing of stress response genes.^5^, ^6^ Moreover, it has become known that beside the positive mechanisms of dissemination there are gene regulation programs to effectuate metastasis suppression, which need to be inactivated for cancers to disseminate.^7^, ^8^ A major advance was achieved, when cancer progression research extended beyond individual genes to gene expression signatures and identified a 4‐pronged core program that is unique to all metastases. Disseminated growths are generally characterized by this program of gene expression, which induces the oxidative metabolism, activates vascularization/tissue remodeling, alters ion homeostasis, and silences extracellular matrix interactions. This signature distinguishes the metastases from their originating primary tumors as well as from their target host tissues.^1^ The discovery has opened the door for the development of anti‐metastasis combination chemotherapies that strive to suppress the components of the core program.

The rationale for the present chemotherapy development is rooted in our research that utilized the gene expression profiles from 653 GEO datasets and a mouse model to investigate whether the signatures by diverse cancers in various metastatic sites display common features.^1^ There is evidence that the gene expression core program of metastasis is activated at the time of release from the primary growth and remains active in the foreign microenvironment of the target site, where continuous survival signals are required. The lasting activity of this genetically encoded core program makes it a suitable target for both, the treatment of existing metastases and the prevention of new metastases.

**FIGURE 1:**
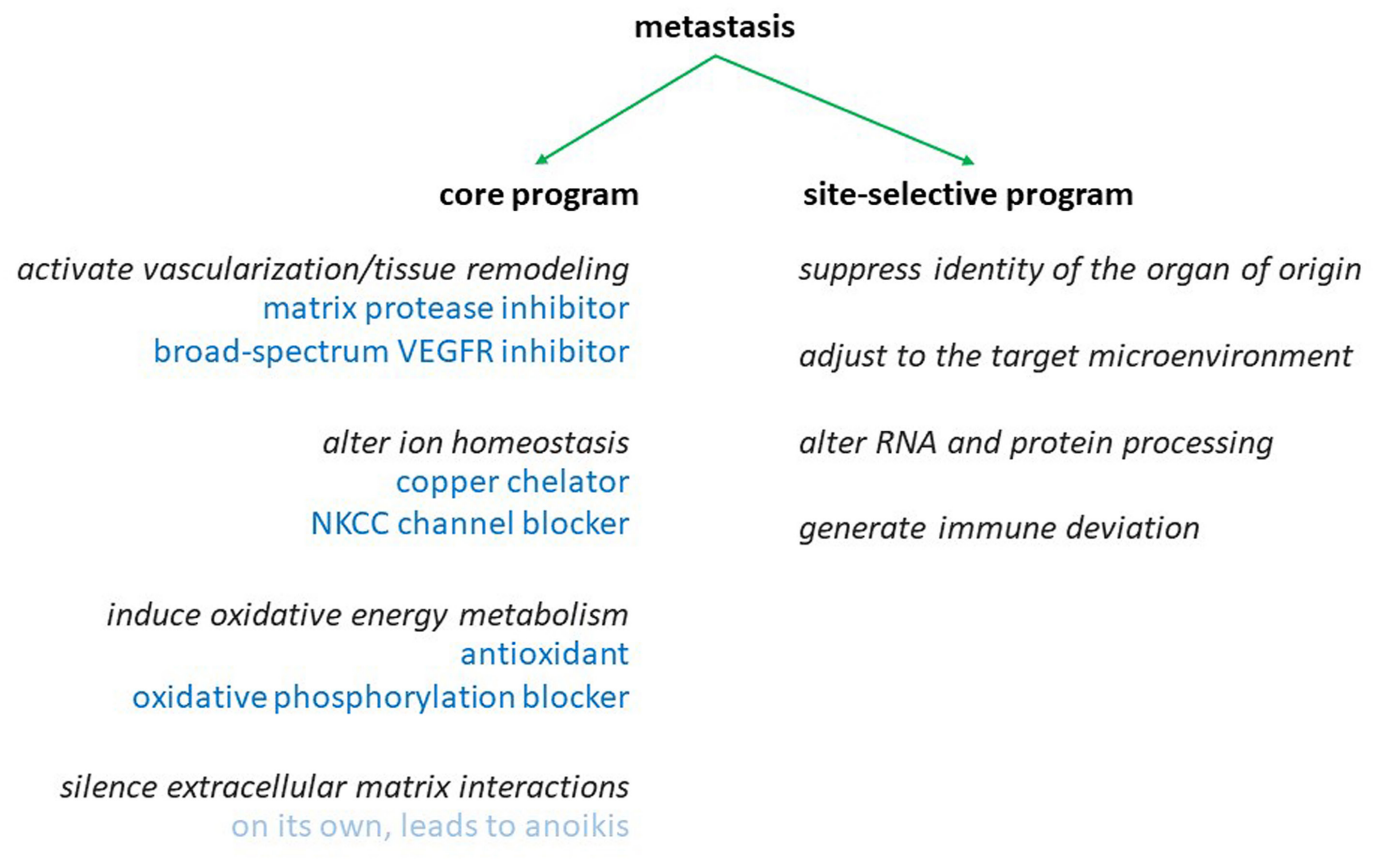
Anti‐metastasis combination chemotherapy. The left panel shows the core program of gene expression in metastasis (black font, from Ref. [1]). Underneath each component, suitable drugs classes are listed in blue font. The right panel displays the site‐selective gene expression program in metastases (from Ref. [2]).

The novelty of this study lies in the buildout of a combination treatment, based on the identification of four pillars in the metastatic gene expression core program (Figure 1). The drug selection criteria entailed the suppression of deadherent cell expansion (a bonified core program component) at doses low enough to not affect adherent cells, the inhibition of soft agar colony formation (because this in vitro assay is a good predictor for in vivo metastatic capability), and increased efficacy when used in drug combination. It was our expectation that the combination chemotherapy to be developed in this manner would be more efficacious (due to addition or synergism) and safer (due to reduced individual drug concentrations) than monotherapy with any of the contributing agents.

In support of prospective clinical translation, the individual candidate agents we are testing here have a record of use in humans, with several of them being approved drugs that are available for repurposing. Previously, various independent literature reports have shown that drugs, which suppress specific components of the core program, can have partial effects, when used individually. Tissue remodeling and angiogenesis were among the first drug targets in this realm. While angiogenesis inhibitors were somewhat efficacious,^9^ the lack of success with metalloproteinase inhibitors in early clinical trials may have been due to suboptimal timing or dosing.^10^, ^11^ In other attempts to counteract metastasis, diverse disruptors of ion homeostasis have been tested. The challenge here has been to identify which ions are the critical ones to support the dissemination of cancer cells and hence can be targeted without adverse effects on the host.^12^ Over time, NKCC transporters and copper have emerged as functionally important, suitable targets. Further, early studies characterized the oxidative metabolism (distinct from the Warburg effect in primary tumors) to be essential for cancer dissemination. The underlying mechanism is an increased requirement for ATP after deadhesion. Paradoxically, literature reports on antioxidants in anti‐metastasis regimens have been conflicting.^13^, ^14^, ^15^, ^16^ While we have found that antioxidant regimens can exert a diversity of effects on cancer dissemination, we reasoned that a properly chosen suppressor of the oxidative metabolism would be required in a combination treatment. Because the silencing of ECM interactions—without added survival signals—may increase the susceptibility to anoikis, we decided to leave it untouched and develop a combination chemotherapy against the other three components.

Unlike oncogenes or tumor suppressor genes, which in cancer are subject to gain‐of‐function or loss‐of‐function mutations, metastasis genes are aberrantly expressed or spliced in malignancies. Therefore, drugs to suppress their mechanism of action do not need to be designed as inhibitors of mutant or altered molecules (although targeting splice variants selectively may be beneficial). Here, we focus on the inhibition of core program drivers that are overactive (aberrantly expressed) in progression and metastasis.

## 2 MATERIALS AND METHODS

### 2.1 Drug selection criteria

It was our premise that a suitable combination chemotherapy can be developed from the ground up. First, we tested drug candidates individually for killing or cell‐cycle‐arresting adhesion‐deprived cells (which represent good models for the cells that have been released from a primary tumor). All agents to be scrutinized have no effect at very low doses; at very high doses, they kill adherent and deadherent cells alike. These drugs would be seen as suitable candidates for further development if they suppressed adhesion‐deprived cells in intermediate, pharmacologic concentration ranges without affecting the same cells in the adherent state (a drug that equally kills adherent and deadherent cells will likely also harm adherent non‐transformed cells; such an agent would not be considered for further development). The initial assessments were performed on cells plated in conventional plastic dishes or on the polymer poly‐HEMA, which prevents adhesion. Second, we then moved to colony formation in soft agar, which depends on a combination of features, including the growth rate of the cells, the expression of gene products for invasiveness, and anti‐anoikis. The ability of cells to form anchorage‐independent colonies in soft agar reflects an important characteristic of transformation and correlates well with their ability to grow invasively in vivo.^17^ We have demonstrated^18^ that benign tumor cells do not form colonies in this assay. By contrast, invasive cancer cells form soft agar colonies under the support from metastasis genes, such as Osteopontin.^19^, ^20^ Third, true anti‐metastasis activity was then tested in murine models.

### 2.2 Cells and drugs

MIA PaCa‐2 cells represent a hypertriploid pancreas carcinoma from a Caucasian male. PANC‐1 are hypertriploid pancreatic epitheloid ductal carcinoma cells from a Caucasian male. MIA PaCa‐2 and PANC‐1 cells were cultured in DMEM 10% FBS (37°C, 5% CO_2_). AsPC‐1 is a metastasis‐derived pancreatic adenocarcinoma from a Caucasian female (RPMI 10% FBS). MDA‐MB‐231 cells are triple negative, aneuploid breast adenocarcinoma cells. They were cultured in DMEM/F12 with 5% FBS. SW1116 (ATCC CCL‐233, DMEM/F12 with 5% FBS) is a colorectal adenocarcinoma. RD are rhabdomyosarcoma cells (ATCC CCL‐136, DMEM with 10% FBS).

Bumetanide, amlodipine, pazopanib, and marimastat were purchased from Selleck Chemicals. Ammonium tetrathiomolybdate, verapamil, and N‐acetyl cysteine (NAC) came from Sigma‐Aldrich. DMSO and taurine were obtained from Fisher Scientific. Atovaquone was received from MyBiosourse.

### 2.3 Deadherent survival

Cells were seeded at a density of 10^4^ cells/well in 96‐well plates, either uncoated or in poly(2‐hydroxyethyl methacrylate) (poly‐HEMA) coated wells (3 mg of poly‐HEMA in 50 μL 95% ethanol, let dry overnight, then washed twice with PBS, followed by one wash with medium before initiation of the experiment). Drugs were added in triplicates at the indicated concentrations for an incubation time of 48 h. At the 48‐h timepoint, the indicator WST‐1 was added to each well at 10% v/v final concentration. At the 60‐, 80‐, and 120‐minute timepoints, the absorbance was measured at 450 nm using a MRX‐TC Revelation plate reader.

### 2.4 Soft agar colony formation

The capability of cells to grow under anchorage‐independent conditions is a hallmark of transformation and correlates with tumor progression in vivo.^17^ The assay was performed in a 60‐mm dish with a bottom layer of 2 mL 0.5% agar (Noble Agar, Sigma) in the culture medium that is designated to the cells. The bottom agar was allowed to be solidified in the biosafety cabinet. Cells were suspended in 0.2% agar for PANC‐1 cells, MIA PaCa‐2 cells, and B16‐F10 cells, but in 0.15% agar for MDA‐MB‐231 cells and 4T1 cells and then plated (1 × 10^5^ cells/2 mL/dish) on top of the bottom layer. 400 μL culture medium with or without the indicated doses of drug treatment was added to cover the top agar layer every other day over 2 weeks for PANC‐1, MIA PaCa‐2 cells and over 3 weeks for MDA‐MB‐231 cells, respectively. The colonies in five microscopic fields (top, bottom, left, right and center) of each dish were photographed. Colony sizes were measured with Image‐J. The colony frequency was found not to be altered, therefore we did not include it in the assessments. Each treatment was repeated thrice.

### 2.5 Confocal microscopy and mitochondrial size assessment

Cells were maintained in soft agar for 5 days. Then, prewarmed (37°C) staining solution containing 100 nM MitoTracker probe (Thermo Fisher Scientific) was added to the dish, followed by incubation for 30 min. The top agar was removed and placed into Nunc Glass Base dishes for visualization under a fluorescence microscope (Leica Stellaris 8 Confocal). The fluorescent areas were photographed and then quantified with Image‐J.

### 2.6 Confocal microscopy and assessment of reactive oxygen species

The redox indicator dye di(acetoxymethyl ester) (6‐carboxy‐2′,7′‐dichlorodihydrofluorescein diacetate) (DCFH‐DA) was obtained from Thermo Fisher Scientific. Cells were maintained in soft agar for 16 h. At that time, they were incubated with 5 μM DCFH‐DA for 30 min. For analysis, we separated the top agar from the bottom agar, before the cellular fluorescence intensity was visualized with confocal microscopy, photographed, and then measured in Image‐J.

### 2.7 In vivo analysis

The in vivo analyses were conducted under an IACUC protocol approved by the University of Cincinnati. Balb/c mice were injected intravenously with 0.2 × 10^6^ 4T1 cells. They were divided into four groups of four to five animals/group, encompassing no treatment (sham injections with PBS), combination chemotherapy (pazopanib + marimastat + DMSO + tetrathiomolybdate + bumetanide), subset treatment 1 (pazopanib + marimastat + DMSO), and subset treatment 2 (tetrathiomolybdate + bumetanide). The dosing was pazopanib 8 mg/kg, marimastat 15 mg/kg, tetrathiomolybdate 5 mg/kg, bumetanide 2 mg/kg, DMSO 200 μL/kg (all in a volume of 150–200 μL, the mice weighed close to 20 g). Treatment was administered every other day, starting either on Day 0 (metastasis prevention) or on Day 5 (treatment of established metastases) up to termination of the experiment at 14 days.

C57Bl/6 mice were injected intravenously (in the tail vein) or subcutaneously (in the flank) with 0.2 × 10^6^ B16‐F10 cells. For the subcutaneous injections, care was taken not to damage the peritoneal lining, The bevel of the needle pointed outward, and the injection angle was as flat as feasible. They were divided into six groups of four animals/group, entailing no treatment (sham injections with PBS), combination chemotherapy (pazopanib + marimastat + DMSO + tetrathiomolybdate + bumetanide), subset treatment 1 leaving out pazopanib, subset treatment 2 leaving out marimastat, subset treatment 3 leaving out tetrathiomolybdate, and subset treatment 4 leaving out bumetanide. The dosing was pazopanib 8 mg/kg, marimastat 15 mg/kg, tetrathiomolybdate 5 mg/kg, bumetanide 2 mg/kg, DMSO 200 μL/kg (in a total volume of 150–200 μL). Delayed treatment added atovaquone at 30 mg/kg. Treatment was administered every other day, either starting on Day 0 (metastasis prevention) or on Day 5 (treatment of established metastases) up to termination of the experiment at 21 days.

At the termination date, the mice were sacrificed, and tissue samples of tumor (after s.c. injection) and lungs (after i.v. injection) were fixed in formalin. Metastasis to the lungs was evaluated qualitatively by photographs and quantitatively by determining metastases counts and lung weights. Abdominal metastases were photographed (where feasible a small portion was saved in RNALater for RNASeq analysis), excised after formalin fixation and weighed for the quantification of tumor burden.

### 2.8 Meta‐analysis

We analyzed NCBI GEO datasets pertaining to cancer metastasis.^1^ Following differential expression analysis of the array data, the identified genes were used as input for the pathway enrichment analysis, which identifies biological pathways (denoted as Gene Ontology terms or GO categories) that are associated with the upregulated or the downregulated genes. An FDR cut‐off of 0.05 was used for selecting significant pathways. Enriched categories (GO terms) were evaluated. The *p*‐value of a GO term was produced using Fisher's exact test (a hypergeometric distribution‐based test), which is very sensitive to the numbers of genes in the up‐ or down‐regulated gene list. From the tables, those categories affecting ion homeostasis according to UniProt were extracted.

### 2.9 Statistics

We used the Wilcoxon–Wilcox test to assess significance in the WST‐1 assays (with corroboration from the Student *t*‐test where indicated) and the Student *t*‐test for paired samples in soft agar and in vivo. ANOVA was applied where indicated. Significance was accepted at the *p* < 0.05 level.

## 3 RESULTS

Three of the four functional categories contained in the metastatic core signature of cancer are promising candidate drug targets (Table 1). The fourth—loss of ECM interaction—may aid therapy once the anti‐anoikis survival mechanisms, imparted by the other three components, are pharmacologically inactivated. We therefore left it untargeted. The drug candidates identified as counteracting tissue remodeling/angiogenesis, oxidative metabolism, and ion homeostasis have in their favor prior records of clinical use (for various conditions).

**TABLE 1:** Candidate drugs to treat three of the four arms in the metastatic core program. The core signature (as reported in^1^) lends itself to drug treatment.

Treatment candidates targeting the metastatic core signature	
*Vascularization/tissue remodeling*	*Low‐specificity VEGFR inhibitor pazopanib*
VEGF in cancer is not limited to angiogenesis and vascular permeability VEGF signaling occurs in tumor cells	Human dose 800 mg per day on empty stomach (initial dose 400 mg, decrease or increase in 200 mg steps based on tolerability) | steady state blood concentration ~20–30 ug/mL | CYP3A4 (major), CYP1A2, and CYP2C8 (minor)
	*Marimastat* (*MMP inhibitor*)
	Inhibitor for MMP‐9, MMP‐1, MMP‐2, MMP‐14, and MMP‐7 with IC_50_ of 3, 5, 6, 9, and 13 nM, respectively | human dose 5–75 mg twice daily or 10–50 mg daily | in vivo 150 mg/kg/day, p.o.
*Oxidative metabolism*	*Hydroxyl radical scavenger DMSO*
	(only topical applications are FDA approved) start with half a teaspoon of DMSO 50% and increase to a teaspoon of DMSO 70%, progress once a week to once a day, only if any possible detoxification reaction is well tolerated; 40% DMSO i.v. has been tried; for prevention of some side effects of cancer treatment, 77%–90% DMSO is applied under medical supervision every 3–8h for 10–14 days | adverse reactions include prolongation of bleeding time, intravascular hemolysis, vision impairment, garlic odor, Herxheimer reactions (detoxification reactions with stomach upset, headaches, dizziness, sedation), kidney and liver function tests are recommended under prolonged use
	*Oxidative metabolism inhibitor atovaquone*
	Inhibits electron transport in mitochondria | 750 mg orally twice a day with food | rash, nausea, vomiting, diarrhea, severe: hepatitis, pancreatitis
	*Peroxide scavenger NAC*
	human nutrition supplement 600 mg per day (drug dose 1200–2400 mg/day, in epilepsy up to 6000 mg/day) | tumor‐bearing mice receive drinking water containing NAC (a 0.001% solution of NAC in water with the pH adjusted to 7.2 by NaOH corresponds to 2 mg/kg/day if 4–5 mL of water are consumed per mouse per day) | steady state plasma concentration ~35 μg/mL (cell culture dose 4 mM) | adverse reactions include rash, nausea and vomiting, angioedema, flushing, tachycardia, bronchospasm, hypotension | deacetylation to L‐cysteine
	*Antioxidant/mitochondrial pH buffer taurine*
	Plasma concentration 50 μM | consumption 40–400 mg/day (but maybe up to 1.5 mg/day)
*Ion homeostasis*	*Bumetanide* (*inhibits the NKCC transporter*)
	Diuretic | usual human daily dosage 0.5–2 mg as a single dose, a second or third dose may be given at 4–5 h intervals up to a maximum daily dose of 10 mg | plasma concentration ~100 ng/mL | 45% unmodified secretion, metabolites by oxidation of the N‐butyl side chain
	*Copper antagonist tetrathiomolybdate*
	Induction: 40 mg 3× per day with meals + 60 mg at bedtime
	Maintenance: total dose in 20 mg increments as needed to maintain copper levels at 5–17 mg/dL
	*Calcium channel blocker amlodipine*
	Oral dose 5–10 mg/day, maintenance: 10 mg/day | long half‐life of 35–50 h | serum concentration ~3–10 ng/mL | side effects include constipation, headache, palpitations, dizziness, rash, drowsiness, flushing, nausea, swelling in the feet | 60% renal elimination, slowly metabolized in the liver by CYP3A4
*Reduction of ECM interactions*	–

### 3.1 Anti‐metastasis effect in vivo

**FIGURE 2:**
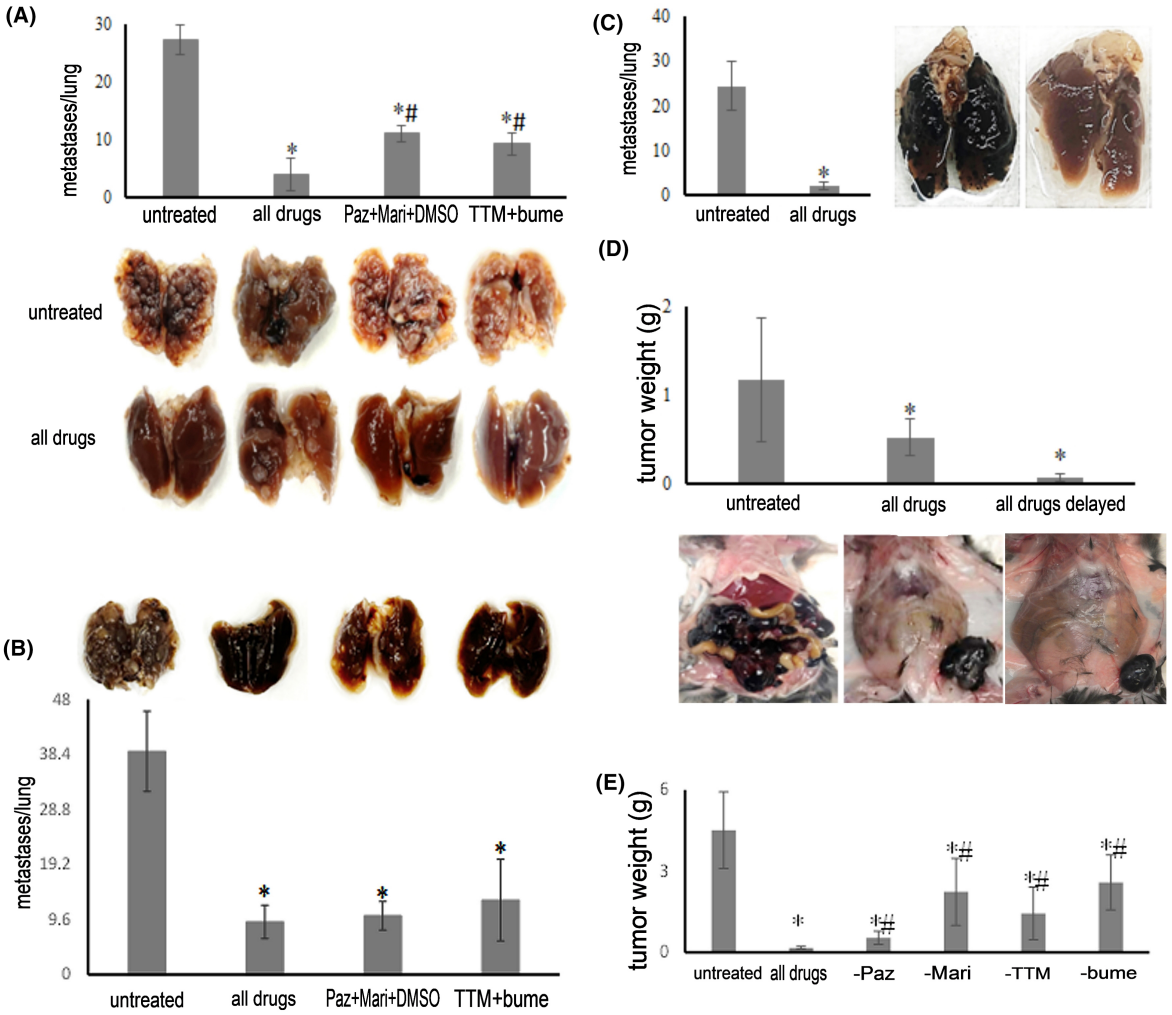
Treatment of metastases in vivo. The dosing was pazopanib 8 mg/kg, marimastat 15 mg/kg, tetrathiomolybdate 5 mg/kg, bumetanide 2 mg/kg, DMSO 200 μL/kg (in a volume of 200 μL). * = significant difference from sham treated, #== significant difference (*p* < 0.05, *t*‐test) from the whole drug combination. (A) Balb/c mice were injected intravenously (tail vein) with 0.2 × 10^6^ 4 T1 cells. Four groups of four animals/group entailed no treatment (sham injections with PBS), combination chemotherapy (pazopanib + marimastat + DMSO + tetrathiomolybdate + bumetanide), subset treatment 1 (pazopanib + marimastat + DMSO), and subset treatment 2 (tetrathiomolybdate + Bumetanide). Treatment was administered every other day, starting on Day 0 up to termination of the experiment at 14 days. The lower panel shows the lungs for the treated and untreated groups. (B) Balb/c mice were injected intravenously (tail vein) with 0.2 × 10^6^ 4 T1 cells. Four groups of four to five animals/group entailed no treatment (untreated: sham injections with PBS), combination chemotherapy (all drugs: pazopanib + marimastat + DMSO + tetrathiomolybdate + bumetanide), subset treatment 1 (Paz + Mari + DMSO: pazopanib + marimastat + DMSO), and subset treatment 2 (TTM + bume: tetrathiomolybdate + Bumetanide). Treatment was administered every other day, starting on Day 5 up to termination of the experiment at 14 days. Above the graph, one representative lung is shown for each treatment group. (C) C57Bl/6 mice were injected intravenously in the tail vein with 0.2 × 10^6^ B16‐F10 cells. Two groups of five animals/group entailed no treatment (untreated: sham injections with PBS) and combination chemotherapy (all drugs: pazopanib + marimastat + DMSO + tetrathiomolybdate + bumetanide). Treatment was administered every other day, starting on Day 0 through the duration of the experiment for 21 days. The panel on the right displays lungs from representative mice after tail vein i.v. injection with sham treatment (left) and the full drug combination (right). (D) C57Bl/6 mice were injected subcutaneously in the left flank with 0.2 × 10^6^ B16‐F10 cells for 21 days. Two groups of five animals/group were injected with combination chemotherapy (pazopanib + marimastat + DMSO + tetrathiomolybdate + bumetanide) from Day 0 (all drugs) and Day 5 (all drugs delayed), one group of five mice entailed no treatment (untreated: sham injections with PBS). The upper panel shows the intraabdominal tumor mass. The lower panel shows representative mice after s.c. injection with sham treatment (left), the immediate full drug combination (middle), and the delayed full drug combination (right). The primary tumor is visible on the left flank of the mice (in the untreated mouse, on the upper right of the picture, because the peritoneal cavity was opened). (E) C57Bl/6 mice were injected subcutaneously in the left flank with 0.2 × 10^6^ B16‐F10 cells. Six groups of four animals/group entailed no treatment (sham injections with PBS), combination chemotherapy (pazopanib + marimastat + DMSO + tetrathiomolybdate + bumetanide), subset treatment 1 leaving out pazopanib, subset treatment 2 leaving out marimastat, subset treatment 3 leaving out tetrathiomolybdate, and subset treatment 4 leaving out bumetanide (Paz, pazopanib; Mari, marimastat; TTM, ammonium tetrathiomolybdate; bume, bumetanide.). The dosing was pazopanib 8 mg/kg, marimastat 15 mg/kg, tetrathiomolybdate 5 mg/kg, bumetanide 2 mg/kg, DMSO 4 μL (in a volume of 200 μL). Treatment was administered every other day, starting on Day 0 up to termination of the experiment at 21 days. The graph displays the total tumor burden of intraperitoneal mass plus primary tumor (in all groups, the primary tumors averaged 0.31 g, with the exception of all drugs at 0.16 g).

We tested the treatment success against in vivo metastases with the drug combination of remodeling inhibitors/antioxidants/ion homeostasis regulators, which had been systematically developed in vitro as described in detail below. Balb/c mice, injected into the tail veins with 0.2 × 10^6^ 4T1 breast cancer cells, develop abundant foci of lung metastases after 14 days. For treatment initiation from the day of injection, the combination of pazopanib + marimastat + DMSO + tetrathiomolybdate + bumetanide achieved 85% suppression of the metastatic burden, with the lungs of two mice being entirely free of macroscopically visible metastases. The treatments with subsets of the full combination therapy (pazopanib + marimastat + DMSO and tetrathiomolybdate + bumetanide) were partially efficacious at 60%–66% inhibition (Figure 2A). In the clinical setting, cancers are often detected only after metastases have formed. Therefore, it is important to test experimental anti‐metastasis treatment for efficacy consecutive to a lag from the injection of tumor cells. Initiating treatment on Day 5 after the intravenous injection of 0.2 × 10^6^ 4 T1 cells and continuing every other day until termination on Day 14 still displayed high efficacy in suppressing the number of disseminated foci on the lungs (Figure 2B).

In preliminary experiments using C57Bl/6 mice, the challenge with i.v. injection of 0.2 × 10^6^ B16‐F10 cells led to abundant lung colonization that was suppressed with the drug combination (Figure 2C). Twenty‐one days after subcutaneous injection in the left flank with 0.2 × 10^6^ B16‐F10 cells, untreated mice (sham injections with PBS) displayed copious abdominal metastases. Under treatment with the combination therapy (pazopanib + marimastat + DMSO + tetrathiomolybdate + bumetanide) from Day 0 (full treatment) or from Day 5 (delayed full treatment), the intraperitoneal dissemination was substantially abrogated compared to the extensive tumor spread in the untreated group (Figure 2D). The treatment with the full combination chemotherapy (pazopanib + marimastat + DMSO + tetrathiomolybdate + bumetanide) completely protected from macroscopically visible metastases. Subset treatment 1 (leaving out pazopanib), subset treatment 2 (leaving out marimastat), subset treatment 3 (leaving out tetrathiomolybdate), and subset treatment 4 (leaving out bumetanide) all had partial effects (Figure 2E). In sum, the drug combination proved highly efficacious with immediate and delayed treatment in two mouse models of cancer metastases.

### 3.2 Target: Tissue remodeling and angiogenesis

**FIGURE 3:**
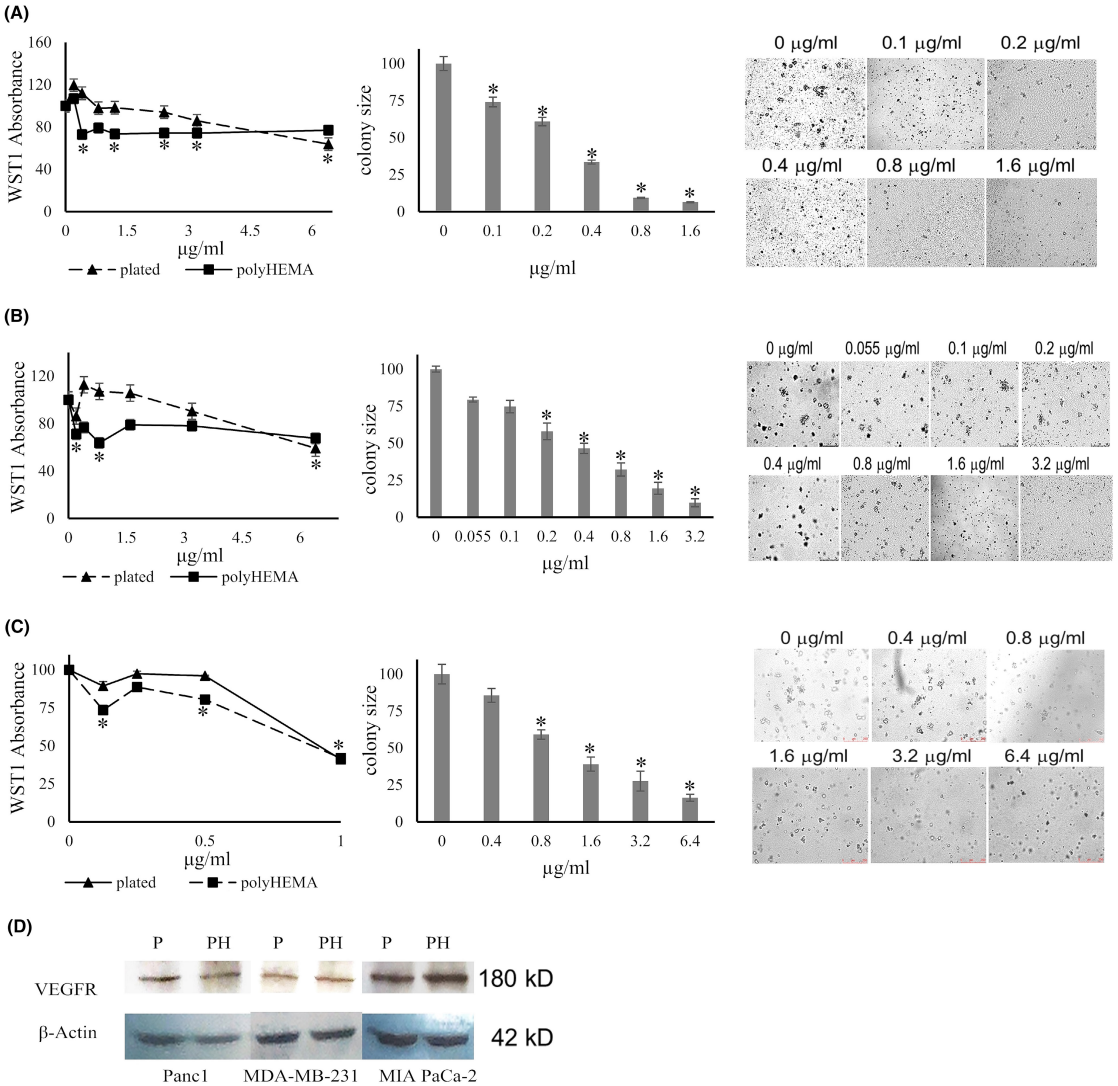
Suppression of anchorage independence by pazopanib. Effect of pazopanib on cell proliferation and soft agar colony formation by MDA‐MB‐231 cells (A), PANC1 cells (B) and MIA Paca‐2 cells (C). (A–C) The left panel shows the proliferation (assessed according to WST‐1 absorbance) of cells plated on poly‐HEMA (squares, solid line) versus plated on the untreated dish (triangles, dashed line) under the titration of pazopanib (*x*‐axis). The middle panel depicts the colony sizes in soft agar under the titration of pazopanib (*x*‐axis). For both assays, the values were normalized to untreated = 100%. Error bars are sem (all data points have error bars, but some are too small to resolve in the graphic display), *indicates significant difference from untreated at *p* < 0.05 (Wilcoxon–Wilcox test for poly‐HEMA assay; the *t*‐test confirmed the results) (*t*‐test for soft agar). The right panel displays representative microscopic fields for MDA‐MB‐231 cells (A), PANC1 cells (B) and MIA Paca‐2 cells (C) at various doses of pazopanib (added every second day). All panels show one representative experiment from at least two. (D) Western blot confirmation of VEGF Receptor‐2 expression as compared to β‐Actin on all 3 cell lines. P, plated; PH, poly‐HEMA. The molecular weights are shown on the right.

Regarding vascularization and tissue remodeling, VEGF signaling is not limited to the blood vessels, but VEGF receptors are also expressed on cancer cells,^21^, ^22^, ^23^ and VEGF signaling occurs. Western blot analysis confirmed VEGFR2 expression in the MDA‐MB‐231, PANC‐1, and MIA PaCa2 cell lines (Figure 3). This expression seemed to be constitutive as it was retained during growth under deadherent conditions. Thus, VEGFR2 is a viable drug target. We focused on broad spectrum (low selectivity) VEGF receptor antagonists^24^ to maximize the odds of blocking the receptor on a wide range of cancer cells.

Furthermore, in situ, tumor‐dependent ECM remodeling is important for dissemination. The secretion of metalloproteinases contributes essentially on this level, therefore rendering these enzymes promising drug targets for inhibitors like marimastat, which is a broad‐spectrum matrix metalloprotease inhibitor for MMP‐9, MMP‐1, MMP‐2, MMP‐14, and MMP‐7. The drug was not tested in the in vitro assays used in this study, because they do not measure metalloproteinase activity. However, marimastat was included in vivo.

#### 3.2.1 Pazopanib inhibits soft agar colony formation by breast and pancreatic cancer cells

We performed soft agar assays to investigate whether pazopanib affects the anchorage‐independent growth by MDA‐MB‐231 cells and PANC‐1 cells (Figure 3). The drug inhibited colony formation in a dose‐dependent manner in both cell lines, reaching significance at 0.5 μM (0.24 μg/mL) in MDA‐MB‐231 cells and at 0.25 μM (0.12 μg/mL) in PANC‐1 cells. It inhibited soft agar colony formation by MIA PaCa‐2 cells at 2.1 μM (1 μg/mL) (but not at 0.1–0.4 μg/mL).

#### 3.2.2 Pazopanib suppresses growth in deadherent breast and pancreatic cancer cells with lesser effects on plated cells

Pazopanib was tested for its growth inhibition of the MDA‐MB‐231 breast cancer cell line, the pancreatic cancer cell line PANC‐1, two additional human pancreatic cancer cell lines (AsPC1, MIA PaCa‐2), and a human colon cancer cell line (SW1116) at 0–6 μg/mL for 48 hours under adherent or deadherent conditions. In all cases, the deadherent cells were more sensitive to the drug effects than the adherent cells (Figure 3). The agent significantly inhibited the growth of MDA‐MB‐231 cells on poly‐HEMA at 0.4–2.0 μg/mL without compromising the same cells in their adherent state. At higher concentrations, both adherent and non‐adherent cells had their WST‐1 uptake suppressed. Pazopanib significantly decreased the cell growth of deadherent PANC‐1 cells at a starting concentration of 0.2 μg/mL, while it increased adherent cell growth at 0.4–2 μg/mL. Above 2 μg/mL, the drug inhibited the growth of PANC‐1 cells under both conditions.

#### 3.2.3 Targeting tissue remodeling with marimastat

The matrix metalloproteinase inhibitor marimastat was not tested in vitro, because our assays do not contain metalloproteinase substrates. However, the drug was added in vivo.

### 3.3 Target: Oxidative metabolism

Deadherent survival and growth require induction of the oxidative metabolism. This may be due to an increased need for ATP once contact to the substratum and to other cells is lost. Distinct from the Warburg effect in primary tumors, increased mitochondrial respiration is needed. There is a growing literature, documenting that peroxides are important contributors to metastasis, and their scavenging suppresses cancer dissemination in murine models.^13^, ^14^, ^25^, ^26^, ^27^, ^28^ We and others^29^ have shown that metastasis and anchorage independence necessitate peroxide signaling. The need for peroxides to support anchorage independence was also confirmed in our research into cancer progression mediated by Osteopontin (which is relevant for about 30 different cancers^30^, ^31^). Osteopontin splice variants induce peroxide signaling in deadherent breast cancer cells, this mechanism is important for survival and expansion, and it is reversible with the antioxidant GSH (or its precursor NAC).^32^ Whereas the full‐length form, Osteopontin‐a, is present in normal and transformed breast tissue, the splice variant Osteopontin‐c is expressed in most breast cancers but not in healthy breasts.^19^, ^33^ While Osteopontin‐a increases the cellular glucose levels, Osteopontin‐c triggers energy generation via peroxide signaling. Each Osteopontin form by itself moderately supports anchorage independence, and their combined effects are synergistic.^29^ The efficacious reversal of anchorage‐independent expansion by genetic or pharmacologic peroxide scavengers (corroborated by the hydrogen peroxide‐mediated support of soft agar colony formation^32^) identifies such agents as anti‐metastasis drug candidates.

#### 3.3.1 Dimethyl sulfoxide (DMSO) inhibits the colony formation of pancreatic cancer cells in vitro

**FIGURE 4:**
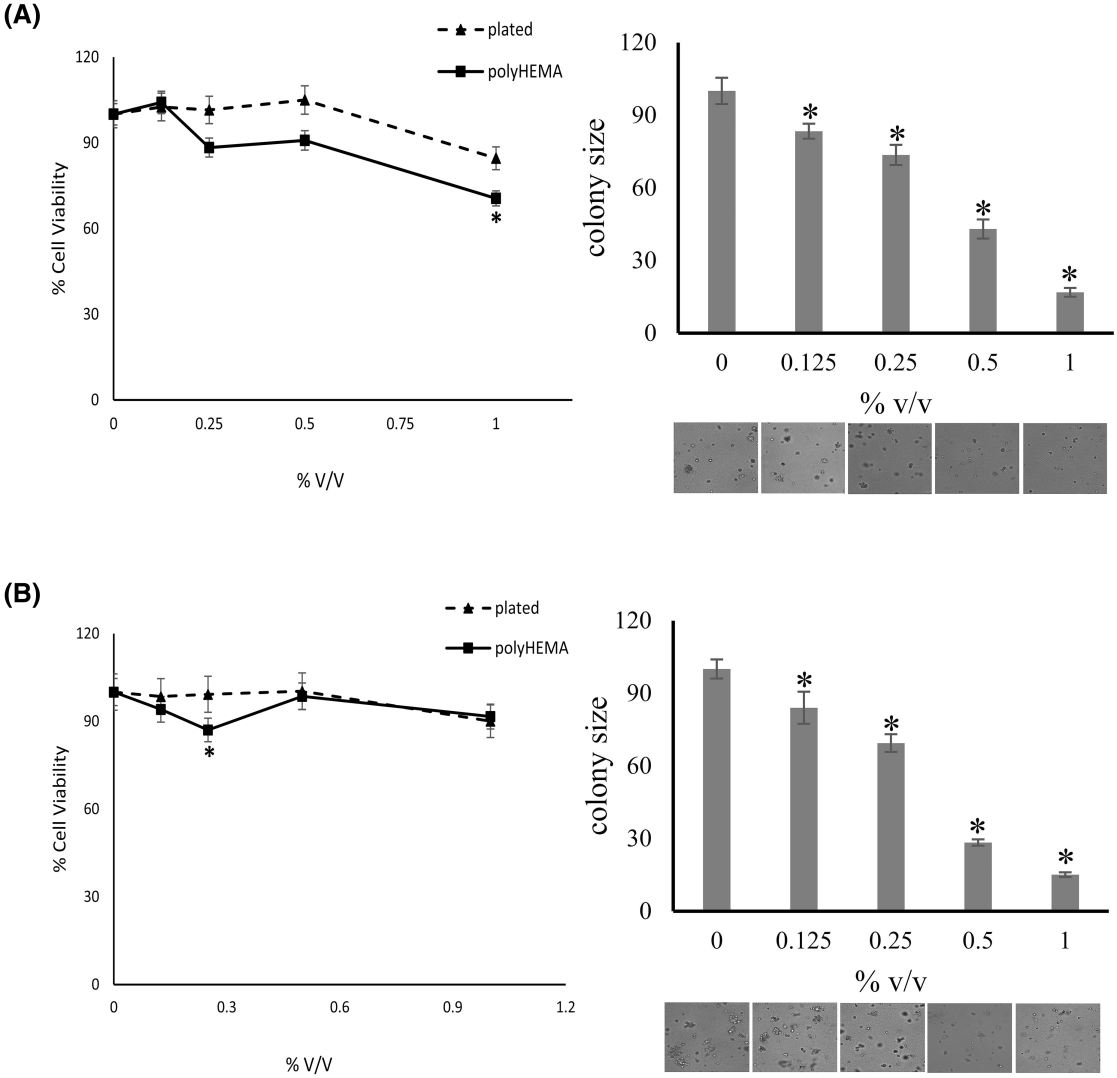
Impact of DMSO on soft agar colony formation. Titration (percent volume/volume) of dimethyl sulfoxide (DMSO) into poly‐HEMA deadhesion assays (left) or soft agar colony formation assays (right) by PANC‐1 cells (A) and MIA PaCa‐2 cells (B). The drug was added at the indicated concentrations every second day with the media replenishment. The values were normalized to untreated = 100%. The error bars are sem, *indicates significant difference from untreated at *p* < 0.05 (Wilcoxon–Wilcox test for poly‐HEMA assay; *t*‐test confirmed for MIA Paca‐2, but showed significance for PANC‐1 poly‐HEMA at and above 0.25%v/v and for plated at the highest DMSO concentration) (*t*‐test for soft agar). The lower panel beneath the soft agar graphs shows representative photographs at the time of evaluation.

Peroxides may react chemically to yield hydroxyl radical. We titrated the hydroxyl radical scavenger DMSO^34^ into soft agar colony formation assays by MIA PaCa‐2 or PANC‐1 cells. In the concentration range of 0.125%–0.25% (v/v), DMSO dose‐dependently suppressed soft agar colony size, without impacting colony frequency (Figure 4).

#### 3.3.2 DMSO does not impact the growth of deadherent or plated cancer cells

In the same concentration range that completely suppressed soft agar colony formation, DMSO had no effect on the growth of PANC‐1, MIA PaCa‐2 or MDA‐MB‐231 cells when plated on plastic or on poly‐HEMA (Figure 4). On further scrutiny, the consistent suppression of soft agar colony formation by DMSO alone or in combination with other agents (see below) prompted us to continue its use.

#### 3.3.3 Atovaquone has promise as an anti‐metastasis agent

**FIGURE 5:**
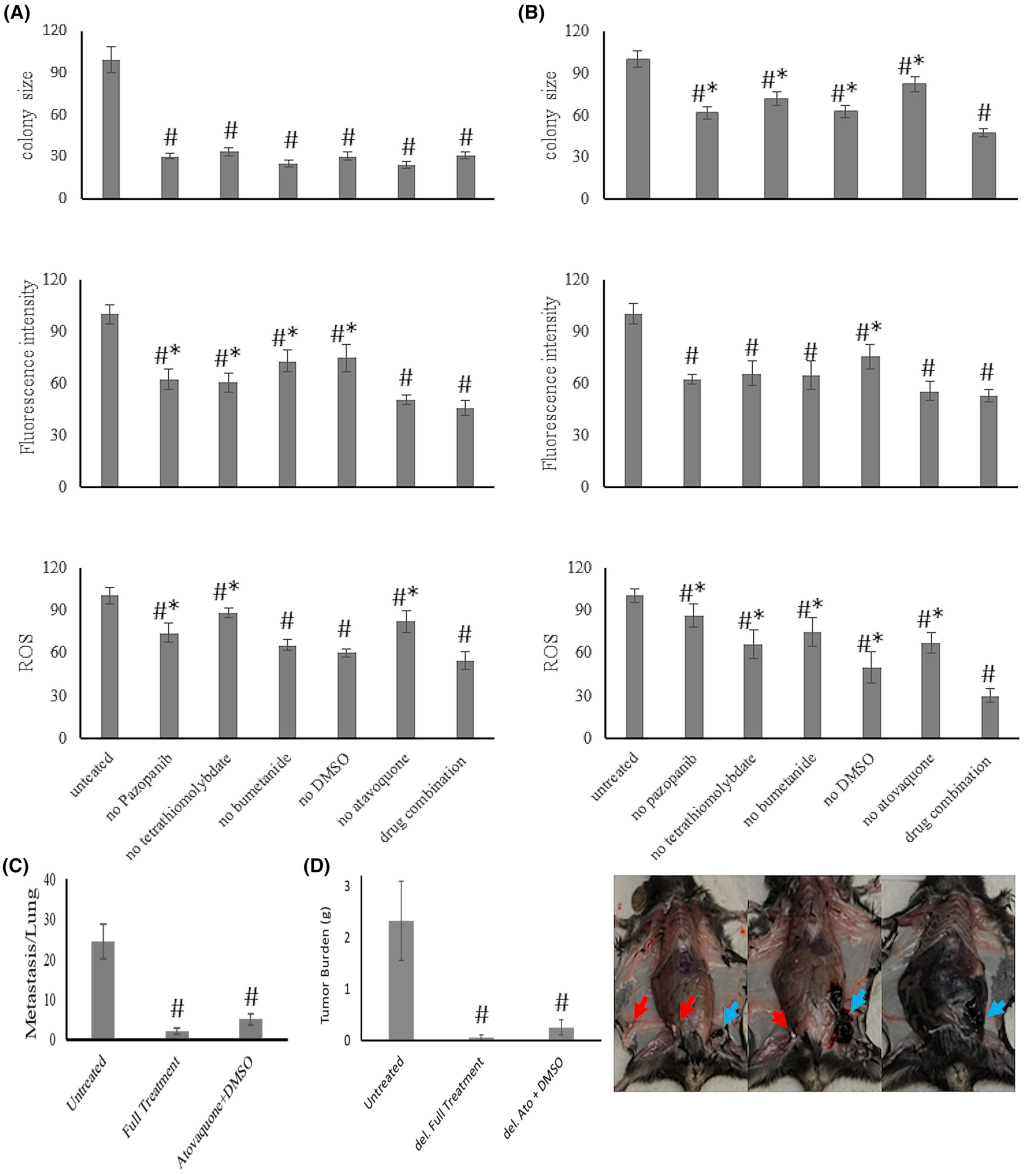
Atovaquone treatment. (A,B) Effects in soft agar. B16‐F10 cells (A) or PANC‐1 cells (B) were plated in soft agar. They were treated every other day with medium alone or with the indicated combinations of pazopanib (0.2 μg/mL), ammonium tetrathiomolybdate (0.25 μg/mL), bumetanide (7.5 μg/mL), dimethyl sulfoxide (DMSO, 0.125%, v/v), atovaquone (5 μg/mL). Colony size was measured after 11 days (top), mitochondrial size (according to a fluorescent dye) after 5 days (middle), and intracellular peroxides (fluorescent indicator) after 16 h (bottom). The values were normalized to untreated = 100%. (C,D) Dissemination after injection of B16‐F10 cells. C57Bl/6 mice were injected with B16‐F10 cells. After 5 days, they were either untreated or treated every second day with the complete drug combination (Full Treatment, pazopanib + ammonium tetrathiomolybdate + bumetanide + DMSO + atovaquone) or DMSO (200 μL/kg) and atovaquone (30 mg/kg). (C) Lung dissemination after intravenous injection. C57Bl/6 mice were injected i.v. with 2 × 10^5^ B16‐F10 cells. The bar graph shows the metastasis count in the lungs after 21 days with or without treatment with atovaquone and DMSO. D) Peritoneal dissemination after subcutaneous injection. C57Bl/6 mice were injected s.c. in the left flank with 2 × 10^5^ B16‐F10 cells. The bar graph shows the intraperitoneal tumor burden (g) of untreated mice compared to mice treated with atovaquone and DMSO (the primary tumors ranged from 0.1 to 0.51 g without differences among the treatment groups). Two representative mice (left and middle) show the primary tumor (blue arrows) and the white specs of apparent drug precipitation (red arrows). A representative untreated mouse (right) has a primary tumor (blue arrow) and abundant dissemination in the peritoneal cavity, which is absent from the treated mice. The error bars are sem. *, significant difference from sham treated; #, significant difference from the whole drug combination (*t*‐test).

Despite its efficacy in preclinical models, DMSO is compromised by lacking approval for internal use in humans. Hence, we sought alternatives. Atovaquone is an oral medication that inhibits oxidative phosphorylation and is FDA approved for the treatment of malaria. It has also demonstrated potential anti‐cancer properties in cell culture. Oxygen consumption and ATP production are inhibited by the drug. This is accompanied by shifts in glycolysis, citric acid cycle, electron transport chain, phospho‐transfer, and metabolism following atovaquone treatment. In mouse models of ovarian and breast cancers, atovaquone previously inhibited the proliferation of cancer cells and ovarian cancer growth in vitro and in vivo.^35^, ^36^, ^37^ These properties make it a good candidate for replacing DMSO (Figure 5).

We tested atovaquone individually and in drug combinations. It suppressed the soft agar colony formation of B16‐F10 cells (Figure 5A) and PANC‐1 cells (Figure 5B) in a dose‐dependent manner. This suppression was correlated to a reduction in intracellular reactive oxygen species according to fluorescence of the redox indicator DCFH‐DA. However, solubility of atovaquone is low (the stock solution was dissolved in DMSO). At the dose used, this resulted in the appearance of crystals in soft agar over time. While the combination of atovaquone and DMSO was efficacious in suppressing metastasis formation by B16‐F10 cells (Figure 5C,D), the appearance of white specs suggested that atovaquone could have formed in vivo precipitates over time. While we believe that this drug will be a beneficial addition to the combination therapy, the solubility concerns became an impediment. There may be potential for including atovaquone in the future (unlike DMSO, it is approved for internal administration to patients), pending further validation.

#### 3.3.4 Other antioxidants have multi‐faceted effects

NAC was efficacious as a monotherapy, when tested in most cell lines. However, when used in combination with other agents, the occurrence of paradoxical exacerbating effects prevented its further use. Endogenous taurine (a mitochondrial antioxidant) is suppressed by the metastasis mediator Osteopontin‐c, but its exogenous supplementation increased soft agar colony formation and precluded its use as well (Supplement 1, Data S1 ).

### 3.4 Target: ion homeostasis

Ion homeostasis is substantially altered in cancer cells^24^ and even more so in metastasis. The choice of promising agents to reestablish ion homeostasis (chelators or channel blockers) was challenging, and the mechanisms of disruption in the ionic balance by metastasizing cells required closer examination. We recently reviewed the literature^12^ to inform on candidate agents. We further revisited our meta‐analysis study^1^ to comprehensively evaluate relevant gene expression profiles of solid tumor metastases retrieved from GEO, where we had performed pathway enrichment analysis (Supplement 2, Data S1).

Ion channel modulators can impact the deadherent survival of cancer cells. The widely reported importance of NKCC transporters, together with early‐stage clinical trials that suggest benefits from copper chelation has put two classes of drug candidates on offer. We tested the effects of cognate antagonists on conventionally plated, adherent cancer cells versus the same cells under deadherent conditions. We also evaluated the inhibition of soft agar colony formation.

#### 3.4.1 Bumetanide inhibits the colony formation of pancreatic cancer cells in soft agar

**FIGURE 6:**
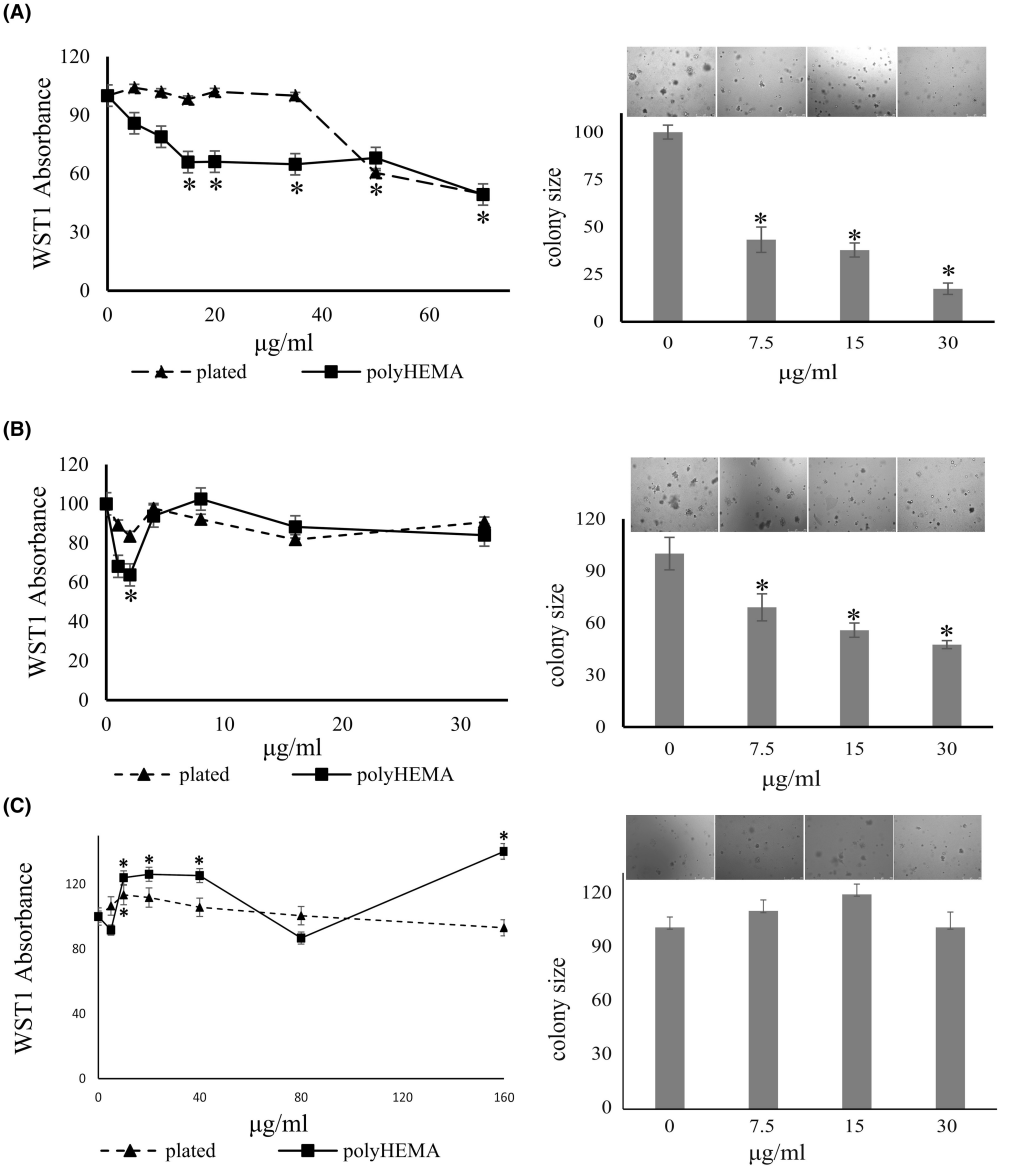
Bumetanide effects on anchorage independence. Shown are the effects by increasing doses of bumetanide on cell proliferation (left panels, poly‐HEMA = squares, solid line; plated = triangles, dashed line) and on soft agar colony formation (right panels, error bars are sem, representative pictures are shown above the bar graphs) by MDA‐MB‐231 cells (A), MIA Paca‐2 cells (B) and PANC‐1 cells (C). All panels show one representative experiment from at least three. The values were normalized to untreated = 100%. *indicates significant difference from untreated at *p* < 0.05 (Wilcoxon–Wilcox test for poly‐HEMA assay, the *t*‐test gave similar results) (*t*‐test for soft agar).

Bumetanide is a specific inhibitor of Na‐K‐2Cl cotransport. The agent significantly decreased the colony formation of MDA‐MB‐231 cells by 56%–82% at a concentration range of 7.5–30 μg/mL (Figure 6). It also suppressed the colony formation of MIA PaCa‐2 cells by 31%–52% at 7.5–30 μg/mL. However, while it suppressed soft agar colony size in all other cells tested, bumetanide did not have any significant effect on the colony formation by PANC‐1 cells. Based on the literature and most of our preliminary data, we decided to hold on to bumetanide in the drug cocktail.

#### 3.4.2 Bumetanide inhibits cell proliferation of deadherent breast cancer cells

The growth of MDA‐MB‐231 cells on poly‐HEMA was inhibited in a dose‐dependent manner by bumetanide treatment, with significant inhibition at 15–35 μg/mL, without affecting plated cells. The drug suppressed both adherent and deadherent cells above 50 μg/mL (Figure 6A). In a concentration range of 1–5 μg/mL, bumetanide suppressed the proliferation of MIA PaCa‐2 cells on poly‐HEMA, but not the plated cells. This differed from the effective range in soft agar colony formation (7.5–30 μg/mL) and was not sustained at higher concentrations. The underlying reasons for the narrow window of inhibition on poly‐HEMA are unknown (Figure 6B).

#### 3.4.3 Ammonium tetrathiomolybdate inhibits the colony formation of cancer cells in soft agar

**FIGURE 7:**
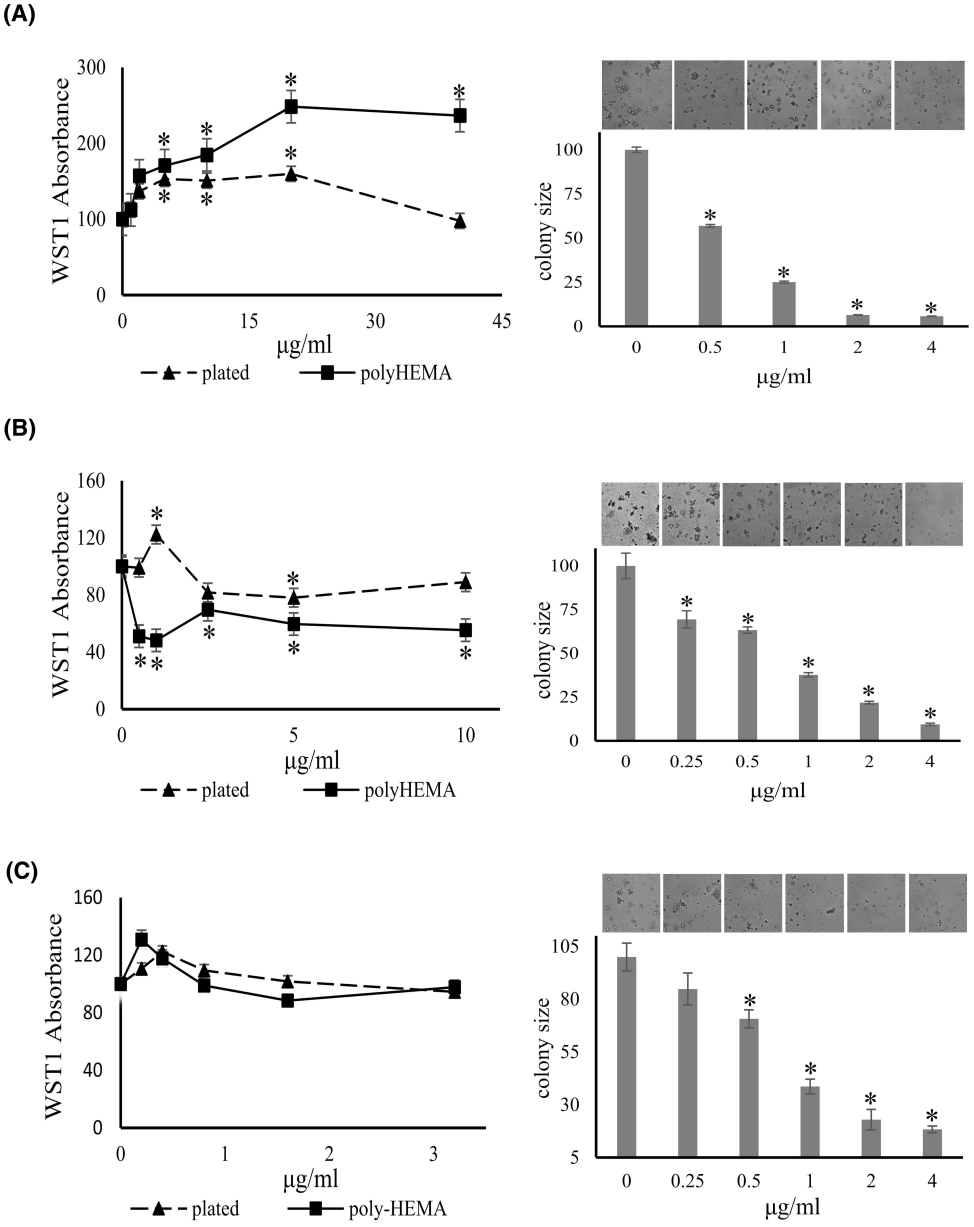
Tetrathiomolybdate effects on anchorage independence. Shown are the effects by increasing doses of ammonium tetrathiomolybdate on cell proliferation (left panels, poly‐HEMA = squares, solid line; plated = triangles, dashed line) and on soft agar colony formation (right panels, error bars are sem, representative pictures are shown above the bar graphs) by MDA‐MB‐231 cells (A), PANC1 cells (B) and MIA Paca‐2 cells (C). The values (one representative experiment from 1 to 4 repeats) were normalized to untreated = 100%. *indicates significant difference from untreated at *p* < 0.05 (Wilcoxon–Wilcox test for poly‐HEMA assay, confirmation with *t*‐test gave similar results) (*t*‐test for soft agar).

Copper is essential for mitochondrial oxidative phosphorylation^38^ and mitochondrial copper depletion suppresses triple‐negative breast cancer in mouse models.^39^ Angiogenesis is required for cancer growth and has become a major target for cancer therapy. Ammonium tetrathiomolybdate is a copper chelator that has been used to treat the neurologic presentations of Wilson's disease.^40^ The agent also has anti‐angiogenic properties.^41^, ^42^, ^43^ We tested the effect of the tetrathiomolybdate salt on soft agar colony formation. The drug inhibited the colony size of MDA‐MB‐231 cells by 43%, 75%, 93%, and 94% at 0.5, 1, 2, and 4 μg/mL, respectively. It similarly inhibited the colony formation of PANC‐1 cells in a dose‐dependent manner, 30%–90% at 0.25–4 μg/mL (Figure 7). Soft agar colony formation by MIA PaCa‐2 cells was suppressed by 33%–54% by 0.25–2 μg/mL ammonium tetrathiomolybdate.

#### 3.4.4 Effects of ammonium tetrathiomolybdate on deadherent cell proliferation

Unexpectedly, ammonium tetrathiomolybdate seemed to increase the growth of MDA‐MB‐231 cells under adherent and deadherent conditions at a concentration range of 0–180 μg/mL (according to increased absorbance of the indicator dye WST‐1). At higher concentrations, the induction of adherent MDA‐MB‐231 cells was inhibited in a concentration‐dependent manner, while the drug moderately increased the absorbance readout by deadherent cells. According to WST‐1 reduction, tetrathiomolybdate significantly suppressed the growth of deadherent PANC‐1 cells, whereas it increased the growth of adherent cells at 0.5–1 μg/mL, then achieved inhibition of both conditions at 2 μg/mL or higher concentrations. The agent modestly increased the cell growth of adherent and deadherent MIA PaCa‐2 cells up to at 0.8 μg/mL (3 μM) (Figure 7). Of note, ammonium tetrathiomolybdate has a color of light orange. It might affect the reading of WST‐1 absorbance, especially at higher concentrations, indicating that there are limitations in applying this cell viability assay. In view of the promising clinical results of the drug against metastatic cancer^38^, ^44^, ^45^, ^46^ and the positive soft agar results, we decided not to dismiss its use.

#### 3.4.5 Calcium channel inhibitors have no selective properties for anti‐metastasis

The drugs amlodipine and verapamil were broadly inhibitory to adherent and deadherent cells alike. They were not deemed suitable for inclusion into the anti‐metastasis drug combination (Supplement 3, Data S1).

### 3.5 Drug combinations

We have validated individual drugs, and we have gained insight into their mechanisms of action (Supplement 4, Data S1) as well as their safety (Supplement 5, Data S1). Combination therapies are intended to increase efficacy (here by targeting multiple mechanisms of metastasis) and reduce toxicity (by allowing dose reduction for each of the component agents). To devise proper drug combinations, we initially selected the agents that had demonstrated efficacy on their own in the in vitro assays (Supplement 6, Data S1). We sought to include two agents for each core program component to be suppressed (comprising pazopanib, marimastat; DMSO, atovaquone; bumetanide, tetrathiomolybdate). As it is impossible to test all dosing permutations for the entire drug combination, initially, the drugs were used at the concentrations that in monotherapy had shown 25% inhibition of soft agar colony formation. To corroborate that each of the component drugs included in the combination treatment actually is an important contributor, we compared the entire cocktail to taking out one drug at a time and comparing the treatment effect in soft agar to untreated as well as fully treated cells. An agent was considered essential if its elimination led to a statistically significant reduction in the suppression of colony formation compared to the drug combination cocktail.

#### 3.5.1 Drug combination effect on cancer cells in soft agar

**FIGURE 8:**
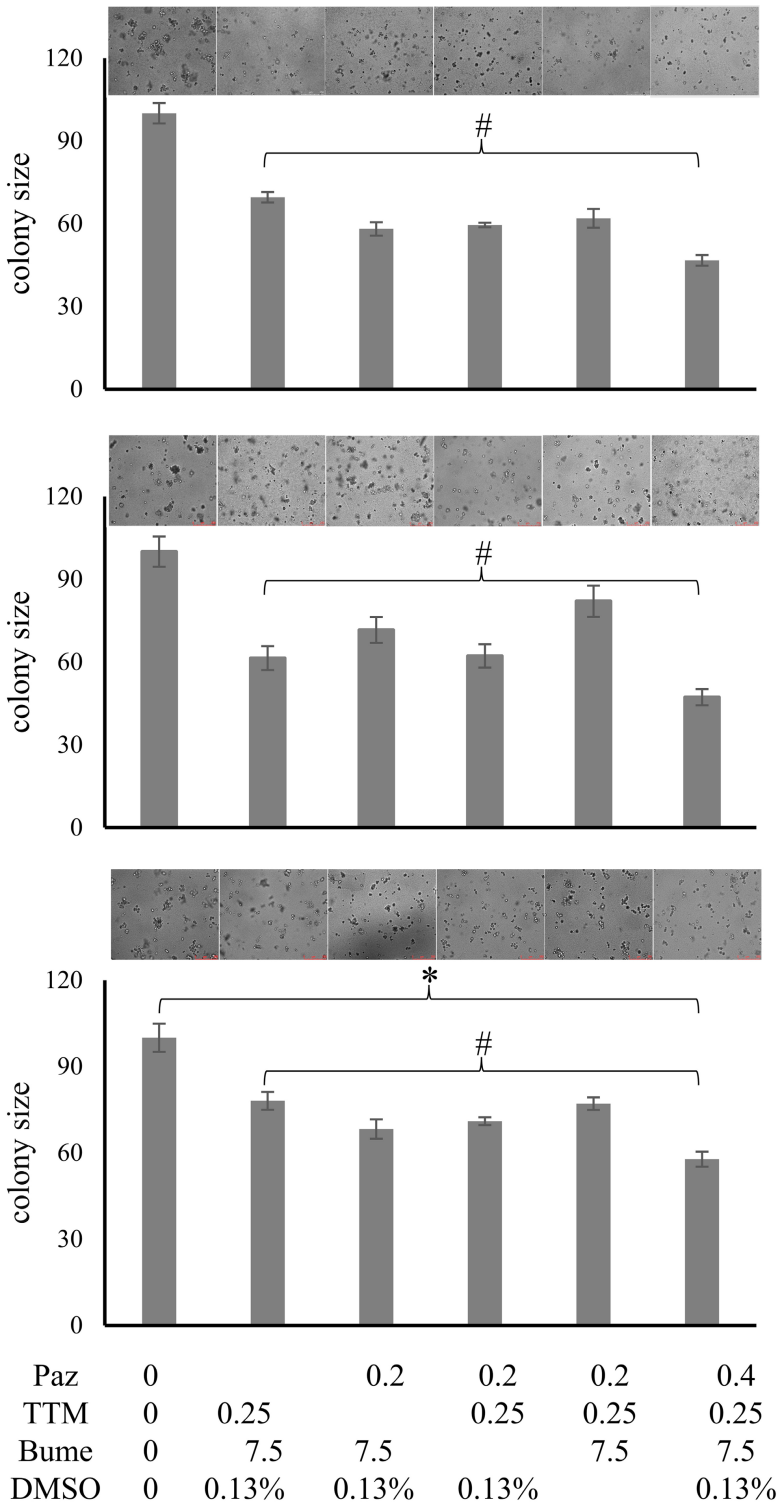
Suppression of soft agar colony formation by drug combinations. The drug combination of pazopanib (Paz), ammonium tetrathiomolybdate (TTM), bumetanide (Bume), and dimethyl sulfoxide (DMSO) was tested for its inhibitory effect on soft agar colony formation by MDA‐MB‐231 cells (top panel), PANC‐1 cells (middle panel), and Mia PaCa‐2 cells (bottom panel) cells. The chosen concentrations represent the 25% percent inhibitory dose individually. The left bar indicates untreated cells, the right bar displays the result in the presence of the full drug cocktail. The bars in between are reflective of the colony sizes when one drug was removed at a time. The error bars are sem. The values were normalized to untreated = 100%. * indicates that all treatments are significantly different from untreated (left bar) at *p* < 0.05. # indicates that all partial treatments were significantly different from the complete combination therapy (right bar) at *p* < 0.05 (ANOVA). Above the bar graphs, representative photographs are displayed of the agars at the time of evaluation.

With the selected drug concentrations (25% inhibition in colony formation individually), we measured the reduction in colony size of MDA‐MB‐231, PANC‐1, and MIA PaCa‐2 cells under combination treatment. To assess the contributions by the individual drugs, we then tested the loss of inhibition in soft agar colony formation upon removal of one agent at a time. According to this analysis, pazopanib, tetrathiomolybdate, bumetanide and DMSO are essential contributors to the suppression of colony formation by the cancer cell lines under study (Figure 8).

## 4 DISCUSSION

The gene expression profile of metastasis is characterized by a core program component and a site‐specific component (see Figure 1). The core program increases vascularization and tissue remodeling/oxidative metabolism/skewing in inorganic ionic balance/motility.^1^ It is activated in various metastases over their respective primary tumors as well as over their target tissues. The ubiquity of this program demanded the testing of drug compounds that inhibit these functions. Here, we have found that 1–2 agents of each, vascularization and tissue remodeling inhibitors, antagonists of the oxidative metabolism, and targeted ionic disruptors are highly efficacious in suppressing metastasis formation and eliminating existing metastases in vivo.

The management of metastatic disease has conventionally been guided by the originating primary tumor, such that liver metastases from colorectal cancer have been treated with drug combinations deemed appropriate for primary colorectal cancer, conversely, liver metastases from pancreatic cancers have been treated with the chemotherapy regimens found suitable for cancers originating in the pancreas. Because the gene expression signatures of cancer metastases evolve substantially from their primary tumors and because metastases from diverse anatomical sites of origin to the same target organ converge in their gene expression patterns, a more favorable strategy could be to focus the selection of combination chemotherapy on the metastases. The rationale is that it may be more efficacious to treat all liver metastases with similar drug regimens that target the genetic core program of metastasis and also the site‐specific genetic program of liver metastasis, regardless of their organ of origin. The chemotherapy of disseminated cancer will be most efficacious if selected to match the genetic makeup of the metastases rather than the organ of origin by the primary tumor. We envision a regimen, in which newly diagnosed cancer patients first receive the anti‐metastasis drug combinations to kill the disseminated cells, before undergoing the treatment considered suitable for eliminating the primary growth (surgically and/or with targeted drugs that suppress the causative gain‐of‐function—mostly neutralizing antibodies and small molecule kinase inhibitors).

Multiple cancer models have been responsive to this novel treatment modality. While we believe that the above three pharmacologic categories always need to be covered in anti‐metastasis combination therapy, there may be some need to exchange individual agents according to tumor type. Whereas all cancers express the core program, they do so by overlapping (albeit tumor‐specifically and target‐specifically distinct) sets of genes. Accordingly, bumetanide suppressed anchorage independence in all cell lines tested, except for PANC‐1. Drugs with similar mode of action include felodipine and ouabain, which are candidates for replacement when treating cancers that resemble PANC‐1. NAC, individually, was highly effective with some cell lines, but not others; and it was potentially exacerbating in combination therapy. Therefore, we replaced NAC with DMSO, which worked reliably. While DMSO has a history of medical use, it is currently not approved for injection or ingestion. The oxidative metabolism inhibitor atovaquone (an antimicrobial, used for the prevention and treatment of Pneumocystis jirovecii pneumonia under the brand name Mepron) also has potential for use, even though its poor solubility became an impediment to its thorough investigation. Resultingly, the basic recommended treatment combination entails pazopanib and marimastat, DMSO and/or atovaquone, bumetanide and ammonium tetrathiomolybdate. Implicit is the possibility to exchange individual drugs for substitutes with comparable mode of action, contingent with their mutual compatibility in pharmacokinetic properties. Further, various modalities of administration are in the realm of possibilities, such as delivery in nanoparticles.^47^, ^48^, ^49^

The chemotherapy of cancers has experienced three major historical milestones. After the introduction of chemotherapy agents around 1946 and some successes with their monotherapy, combination therapy (introduced in 1965 and refined in countless clinical trials through the end of the century) achieved substantial reductions in toxicity. The molecular biology research that had been conducted in cancer since 1976 came to fruition as the third milestone around 1997–2000 with the introduction of new‐generation anti‐cancer drugs (neutralizing antibodies and small molecule kinase inhibitors), which specifically target the mutations that are causative for individual cancers.^24^ Many of these targeted drugs finally achieved a treatment focus on qualitative differences between tumors and host tissues (e.g., BCR‐ABL for imatinib, kinase mutations in EGFR for gefitinib), to the substantial gain of efficacy and safety. Since then, progress has been achieved in targeting tumor‐host interactions (hormone therapy, anti‐angiogenesis, immunotherapy). However, metastasis has remained undertreated. Our report introduces a novel combination regimen to fill this void. Prima facie, therapy modalities that utilize agents, which are not specific for mutated molecules in cancer cells (as introduced here for anti‐metastasis drugs), may seem like a suboptimal choice. Contrarily, we believe that specificity is gained in the anti‐metastasis regimens reported here through their combined effects. Individually, these agents are only modestly impactful on healthy adult tissues. On the basis of the unique characteristics of the metastasis‐associated genetic programs, the synergistic effect of the drug combination is sufficiently selectively exerted on the disseminating cells and the disseminated foci.

Due to the difficulty of eliminating cancer cells, multiple 5‐drug combinations have been used in oncology. They include BEACOPP (a chemotherapy regimen for the treatment of Hodgkin's lymphoma), VAPEC‐B (for high grade non‐Hodgkin lymphoma), PMit‐CEBO (for non‐Hodgkin's lymphoma), ChlVPP/EVA hybrid (for Hodgkin's lymphoma), MOPP/EBV/CAD hybrid (for Hodgkin's disease), Stanford V (for advanced Hodgkin's lymphoma), C‐VAMP (for Hodgkin's lymphoma in children), and CMFVP (for breast cancer). Mostly, those were empirically found after numerous clinical trials. By contrast, the present combination has been rationally derived from the discovery of a metastasis core program. Several of the component drugs have been studied in trials individually, but understandably have shown limited efficacy (Supplement 7, Data S1). Their combination may very well achieve dramatic improvements in the fight against metastases.

## AUTHOR CONTRIBUTIONS

**Gulimirerouzi Fnu:** Data curation (equal); formal analysis (equal); writing – original draft (equal); writing – review and editing (equal). **Georg F. Weber:** Conceptualization (equal); formal analysis (equal); funding acquisition (equal); methodology (equal); validation (equal); writing – original draft (equal); writing – review and editing (equal).

## CONFLICT OF INTEREST STATEMENT

The authors declare no potential conflicts of interest.

## Supporting information


Data S1.

## Data Availability

Data are available upon request.
