# Supplementary material for: Targeting the core program of metastasis with a novel drug combination
Source: Cancer Med. 2024 Jun 3;13(11):e7291. doi: 10.1002/cam4.7291 (PMC11145026; doi:10.1002/cam4.7291)
Supplement: Supplementary file 1 — Data S1. [file CAM4-13-e7291-s001.pdf]

Supplementary Material for

Targeting the Core Program of Metastasis with a Novel Drug Combination

Gulimirerouzi Fnu[1], Georg F. Weber[1*]

[1] University of Cincinnati Academic Health Center, Cincinnati, OH, USA

Supplement 1: Antioxidants

Supplement 2: Ion homeostasis

Supplement 3: Calcium channel blockers

Supplement 4: Mechanism of action

Supplement 5: Drug safety

Supplement 6: Summary of drug actions

Supplement 7: Clinical trials of individual component drugs

Supplement 1: Antioxidants

Drug candidate NAC. Metastasis requires peroxide signaling (29), and we have experimental results (32) demonstrating the effect of the peroxide scavenger NAC on suppressing anchorage-independent expansion. There are conflicting accounts in the literature (16,15), but we believed that previously reported adverse results may have reflected inadequate dosing (also discussed in (29)). NAC has been proven safe in other clinical applications, even at very high doses. It serves as a precursor for glutathione, which is present inside human cells at millimolar concentrations (50,51).

NAC inhibits the colony formation of breast and pancreatic cancer cells in vitro. We evaluated NAC for its activity on anchorage-independence in the MDA-MB-231 breast cancer and PANC-1, MIA PaCa-2 pancreatic cancer cell lines. The agent inhibited the cell colony formation by both MDA-MB-231 and PANC-1 cell lines in a concentration-dependent manner up to 4 mM (Figure S1A,B), whereas it did not affect the colony formation of MIA PaCa-2 cells at the given concentrations (not shown). To ensure our system is consistent with prior results, we confirmed the previously published NAC effects on MCF-7 OPNc colony formation in soft agar (32). Our results are in agreement with the reported outcomes, where NAC suppressed the anchorage-independent expansion of MCF-7 transfectants (Figure S1C).

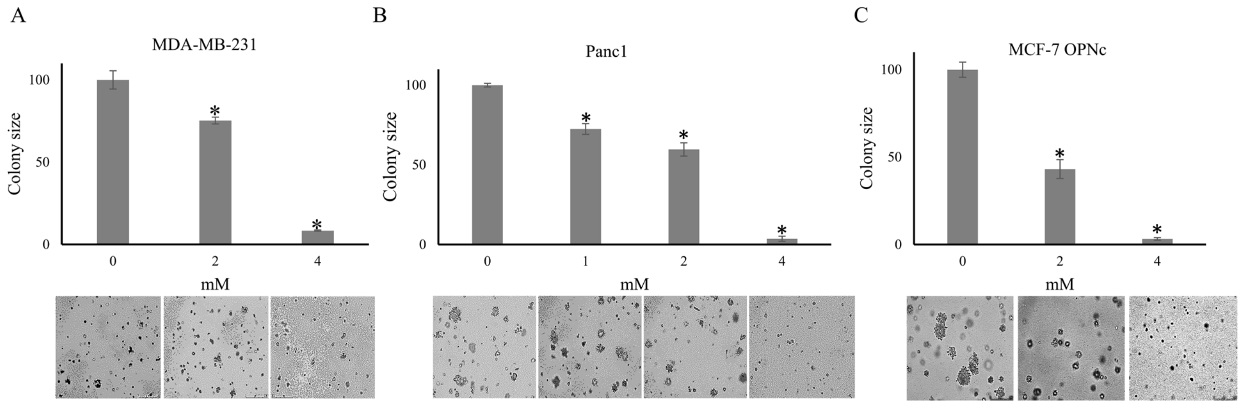

Figure S1: Impact of NAC on soft agar colony formation. A,B) NAC, added every other day at the indicated final concentrations, suppresses the colony formation on MDA-MB-231 cells (A) and PANC-1 cells (B) in a concentration-dependent manner. **C)** NAC confirmation in MCF-7 cells stably transfected with Osteopontin splice variant -c (OPNc). * indicates significant difference from the untreated control at the $p < 0.05$ level. The values were normalized to untreated = 100%.

Of note, we could not assess NAC in the WST-1 assay. NAC changes the medium color, which affects the absorbance reading. This skewing can be controlled by adding NAC to wells

without cells. Worse, the WST-1 assay is based on a redox reaction. NAC may reduce the color reagent directly.

Unexpectedly, when NAC was added to combination treatments of the tissue remodeling inhibitor pazopanib, the ionic disruptors bumetanide and ammonium tetrathiomolybdate, and the antioxidant dimethyl sulfoxide, NAC consistently reduced the efficacy of the drug treatment in MDA-MB-231 cells, PANC-1 cells, as well as MIA PaCa-2 cells (Figure S1D). Hence, yet unexpained drug-drug interactions caused a paradoxical effect and prevented to use of NAC in the anti-metastasis combination treatment.

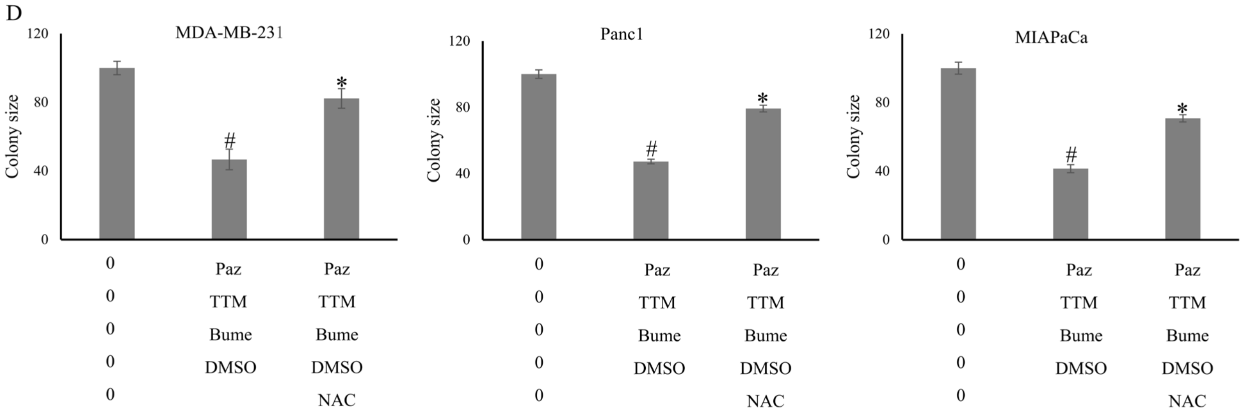

Figure S1: NAC in combination treatments. D) Displayed are the soft agar colony sizes without treatment, under treatment with 0.2 μg/ml pazopanib (Paz)/0.25 μg/ml tetrathiomolybdate (TTM)/7.5 μg/ml bumetanide (Bume)/0.125% dimethylsulfoxide (DMSO), and under the same drug combination plus the addition of 2 mM N-acetyl cysteine (NAC). # = significant reduction in colony size compared to untreated, * = significant increase in colony size compared to the drug combination without NAC.

Drug candidate taurine. Taurine is a mitochondrial antioxidant. It was previously shown to be suppressed by the metastasis mediator Osteopontin-c (32). This suggested that exogenous taurine supplementation could serve as an antioxidant treatment (at least for hypochlorite, which it detoxifies to *N*-chlorotaurine (52)). However, adding taurine to soft agar assays at 0.5-3.5 mM resulted in larger, not smaller colonies by PANC-1 and MCF-7 OPNc cells (Figure S1E), and it elevated the intracellular levels of reactive oxygen species in PANC-1 cells as assessed with the fluorescent dye di(acetoxymethyl ester)(6-carboxy-2',7'-dichlorodihydrofluorescein diacetate) (Figure S1F). Taurine also did not convey a benefit in combination with other drugs (Figure S1G).

Figure S1: Taurine is not a drug candidate. E) Soft agar colony size under treatment with the indicated increasing doses of taurine. **F)** Fluorescence measurement of the cellular redox state under treatment with taurine. PANC-1 cells were grown in soft agar for 16 hours. Measurement

of intracellular reactive oxygen species according to di(acetoxymethyl ester)(6-carboxy-2',7'-dichlorodihydrofluorescein diacetate) fluorescence. **G)** Taurine in drug combinations. Displayed are the soft agar colony sizes without treatment, under treatment with 0.2 μg/ml pazopanib (Paz)/0.25 μg/ml tetrathiomolybdate (TTM)/7.5 μg/ml bumetanide (Bume), and under the same drug combination plus the addition of 400 μM taurine. # = significant reduction in colony size compared to untreated, * = significant increase in colony size compared to the drug combination without NAC. The values were normalized to untreated = 100%.

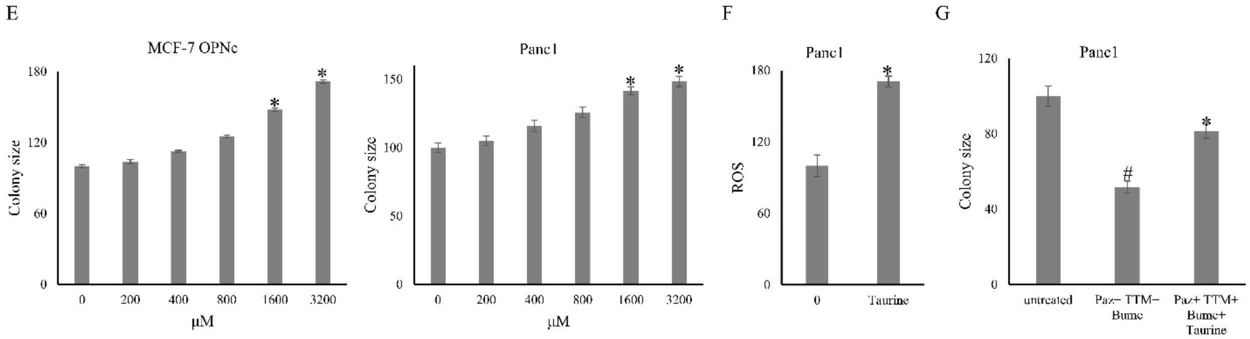

Reactive oxygen intermediates possess a broad range of anti- and pro-tumorigenic roles (29,53), which have complicated the decision-making, when to treat with antioxidants and if so, which ones. While there has been little doubt that deadhesion increases peroxides in cancer cells, there have been conflicting experimental observations whether these peroxides exert limiting or exacerbating effects on metastasis formation (16,54-56). The results obtained here highlight the delicate balance of oxidants and antioxidants in transformed cells. An agent such as NAC, which individually suppresses anchorage-independent survival and growth in a number of transformed cells, can assume aggravating properties in combination with other agents. Whereas endogenous taurine has antioxidant properties, its exogenous addition increases the intracellular peroxide levels and promotes anchorage-independent growth.

Supplement 2: Ion homeostasis

We revisited our meta-analysis study (1) to comprehensively evaluate relevant gene expression profiles of solid tumor metastases retrieved from GEO, where we had performed pathway enrichment analysis (Figure S2). When considering all associations with ion homeostasis that are captured in relevant GO categories (including ion transport, ion binding, ionic cofactor requirements, and contributions in biological responses to ionic agents), the results were broad. Calcium and zinc emerged as strongly affected in disseminated tumor cells; sodium, potassium and chloride also displayed as frequently deregulated.

Figure S2: Gene ontology pathways altered in various metastatic sites. Among the top 100 changes in gene expression pathways within the groups of metastases compared to the host organ (A), compared to the primary tumor (B), or derived from a murine model (C), gene ontology categories that reflect components of the inorganic ion transport and homeostasis are shown.

A) metastases compared to the host organ

																					up versus target
**liver**			**lung**			**bone**			**ovary**			**peritoneum**			**adrenal**			**lymph node**			
**term**	**p-value**	**FDR**	**Term**	**P value**	**FDR**	**Term**	**P value**	**FDR**	**Term**	**P value**	**FDR**	**Term**	**P value**	**FDR**	**Term**	**P value**	**FDR**	**Term**	**P value**	**FDR**	
			GO:0000287~magnesium ion binding	0.047	0.213																
									GO:0010035~response to inorganic substance	0.001	0.013	GO:0010035~response to inorganic substance	0.002	0.022							
GO:0015296~anion:cation symporter activity	0.002	0.023																			
						GO:0015985~energy coupled proton transport, down electrochemical gradient	0.000	0.000													
						GO:0016469~proton-transporting two-sector ATPase complex	0.000	0.000													
						GO:0016820~hydrolase activity, catalyzing transmembrane movement	0.000	0.000													
						GO:0019829~cation-transporting ATPase activity	0.000	0.000													
						GO:0034220~ion transmembrane transport	0.000	0.000													
															GO:0034704~calcium channel complex	0.000	0.002				
						GO:0042625~ATPase activity, coupled to transmembrane movement of ions	0.000	0.000													
						GO:0042626~ATPase activity, coupled to transmembrane movement of substances	0.000	0.000													
						GO:0043492~ATPase activity, coupled to movement of substances	0.000	0.000													
						GO:0046961~proton-transporting ATPase activity	0.000	0.001													
																					down versus target
**liver**			**lung**			**bone**			**ovary**			**peritonum**			**adrenal**			**lymph node**			
**term**	**p-value**	**FDR**	**term**	**p-value**	**FDR**	**term**	**p-value**	**FDR**	**term**	**p-value**	**FDR**	**term**	**p-value**	**FDR**	**term**	**p-value**	**FDR**	**term**	**p-value**	**FDR**	
																		GO:0005216~ion channel activity	0.115	0.298	
			GO:0005275~amine transmembrane transporter	0.005	0.113																
																		GO:0005506~iron ion binding	0.055	0.220	
												GO:0006818~hydrogen transport	0.000	0.003							
			GO:0006820~anion transport	0.004	0.109	GO:0006820~anion transport	0.000	0.003										GO:0006820~anion transport	0.007	0.095	
												GO:0006873~cellular ion homeostasis	0.000	0.003				GO:0006873~cellular ion homeostasis	0.016	0.141	
			GO:0008509~anion transmembrane transporter	0.028	0.207	GO:0008509~anion transmembrane transporter	0.001	0.008	GO:0008509~anion transmembrane transporter	0.005	0.054							GO:0008509~anion transmembrane transporter	0.002	0.083	
															GO:0009055~electron carrier activity	0.001	0.008	GO:0009055~electron carrier activity	0.073	0.259	
												GO:0009309~amine biosynthetic process	0.000	0.002							
			GO:0010035~response to inorganic substance	0.017	0.169	GO:0010035~response to inorganic substance	0.001	0.008													
																		GO:0015276~ligand-gated ion channel activity	0.003	0.083	
			GO:0015294~solute:cation symporter activity	0.009	0.143	GO:0015294~solute:cation symporter activity	0.000	0.000													
						GO:0015296~anion:cation symporter activity	0.000	0.000													
						GO:0015370~solute:sodium symporter activity	0.000	0.003													
						GO:0051536~iron-sulfur cluster binding	0.000	0.005													
						GO:0051540~metal cluster binding	0.000	0.005													
																		GO:0015674~di-, tri-valent inorganic cation transport	0.009	0.109	
			GO:0015698~inorganic anion transport	0.020	0.177																
												GO:0015985~energy coupled proton transport	0.000	0.001							
												GO:0016469~proton-transporting ATPase complex	0.000	0.001							
												GO:0016820~hydrolase activity, catalyzing transmembrane movement	0.000	0.002							
GO:0019220~regulation of phosphate metabolism	0.000	0.005																			
												GO:0019829~cation-transporting ATPase activity	0.000	0.000							
																		GO:0022834~ligand-gated channel activity	0.003	0.083	
																		GO:0022836~gated channel activity	0.029	0.164	
																		GO:0022838~substrate specific channel activity	0.136	0.329	
																		GO:0030001~metal ion transport	0.085	0.269	
																		GO:0030003~cellular cation homeostasis	0.077	0.262	
												GO:0033178~proton-transporting ATPase complex	0.000	0.000							
												GO:0034220~ion transmembrane transport	0.000	0.000							
												GO:0046961~proton-transporting ATPase activity	0.000	0.000							
																		GO:0050801~ion homeostasis	0.004	0.083	
																		GO:0055066~di-, tri-valent inorganic cation homeostasis	0.111	0.294	
																		GO:0055080~cation homeostasis	0.038	0.178	

B) metastases compared to the primary tumor

												up verus source
**breast**				**kidney**				**prostate**				
**Term**	**P value**	**FDR**		**Term**	**P value**	**FDR**		**Term**	**P value**	**FDR**		
				GO:0005249~voltage-gated potassium channel activity	0.00568521	0.03675						
				GO:0006813~potassium ion transport	0.00704481	0.04152						
				GO:0006873~cellular ion homeostasis	0.00310905	0.02703						
				GO:0008076~voltage-gated potassium channel complex	0.00850924	0.04748						
GO:0010035~response to inorganic substance	3.4E-03	0.03824										
				GO:0030955~potassium ion binding	0.00341914	0.02873						
				GO:0034705~potassium channel complex	0.00850924	0.04748						
				GO:0050801~ion homeostasis	0.00443806	0.03227						
												down versus source
**breast**				**kidney**				**prostate**				
**Term**	**P value**	**FDR**		**Term**	**P value**	**FDR**		**Term**	**P value**	**FDR**		
GO:0006873~cellular ion homeostasis	4.7E-04	4.8E-03						GO:0006873~cellular ion homeostasis	5.9E-04	6.0E-03		
				GO:0010035~response to inorganic substance	0.00442631	0.0404		GO:0010035~response to inorganic substance	8.4E-05	1.8E-03		
				GO:0015294~solute:cation symporter activity	0.00793226	0.05369						
				GO:0015370~solute:sodium symporter activity	0.01121766	0.06423						
								GO:0030003~cellular cation homeostasis	0.00118277	0.00963		
GO:0050801~ion homeostasis	1.7E-04	2.7E-03						GO:0050801~ion homeostasis	0.000858693	0.00787		
GO:0055065~metal ion homeostasis	8.5E-04	7.1E-03						GO:0055065~metal ion homeostasis	0.001776101	0.0122		
								GO:0055080~cation homeostasis	0.001566596	0.01155		

C) mouse model of metastasis

**mets vs. primary up**		**mets vs. primary down**		**mets vs. host up**		**mets vs. host down**
				GO:0010043 / response to zinc ion		
				GO:0046686 / response to cadmium ion		
				GO:0071248 / cellular response to metal ion		
				GO:0071276 / cellular response to cadmium ion		
				GO:0071294 / cellular response to zinc ion		
				GO:1990267 / response to transition metal nanoparticle		

Supplement 3: Calcium channel blockers

Effects of calcium channel blockers on adherent and deadherent cells. We investigated the effects of the calcium channel blockers amlodipine and verapamil on soft agar colony formation and on the adherent/deadherent proliferation of MDA-MB-231 breast cancer cells as well as pancreatic cancer cell lines (PANC-1 and MIA PaCa-2). Amlodipine and verapamil both affected adherent and deadherent cells similarly (Figure S3). This lack of selectivity by calcium channel blockers for deadherent cells is consistent with their reported adverse effects in cancer. Long-term use of calcium-channel blockers is associated with a greater than 2-fold increase in the risk for breast cancer in postmenopausal women (57). Calcium channel blockers, specifically the short-acting forms, appear associated with an increased risk for pancreatic cancer among postmenopausal women (58). We did not pursue their use.

Drug candidate amlodipine. Adherent MDA-MB-231 cells were more sensitive to amlodipine than deadherent cells. At treatment concentrations of above 10 μM, amlodipine significantly inhibited adherent cell growth and achieved full inhibition at 80 μM. At 0-40 μM concentrations, the drug did not compromise the deadherent cell growth (Figure S3A). Amlodipine significantly inhibited MIA PaCa-2 cells under both adherent and deadherent conditions in a dose-dependent manner and achieved full inhibition at 15 μg/ml and 20 μg/ml, respectively (not shown). PANC-1 cells reacted slightly differently to amlodipine treatment, with an increased growth of deadherent cells over a concentration range up to 20 μg/ml (at which it fully inhibited adherent cells), before complete inhibition under both conditions at 30 μg/ml (not shown). Amlodipine significantly decreased the colony formation of MDA-MB-231 cells at 5 μM and 10 μM (p 0.001) and fully inhibited colony formation of MDA-MB-231 and PANC-1 cells at 20 μM (Figure S3B,C).

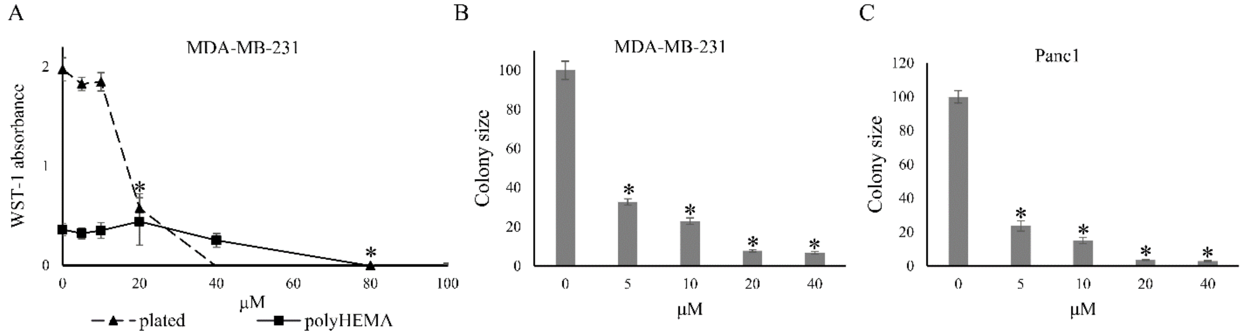

Figure S3: Amlodipine effects on cell proliferation and soft agar colony formation. A) Titration of amlodipine to MDA-MB-231 cells on poly-HEMA (squares, solid line) and plated (triangles, dashed line). **B,C)** Amlodipine-dependent suppression of soft agar colony formation by MDA-MB-231 cells (B) and PANC-1 cells (C). The values were normalized to untreated = 100%. * indicates significance at $p < 0.05$.

<u>Drug candidate verapamil.</u> Verapamil increased the cell growth of adherent MDA-MB-231 cells at 0-60 μM, then showed dose-dependent progressive inhibition, and full inhibition occurred at 200 μM. The drug failed to affect deadherent cells up to 100 μM, before inhibition occurred at 100-400 μM (Figure S3D). Likewise, verapamil increased the viability of adherent PANC-1 and MIA PaCa-2 cells up to 10 μg/ml and 0-20 μg/ml, respectively. Then it caused dose-dependent inhibition at higher concentrations (not shown). Verapamil did significantly inhibit soft agar colony formation of MDA-MB-231 cells at 20 μM (p<0.001) (Figure S3E).

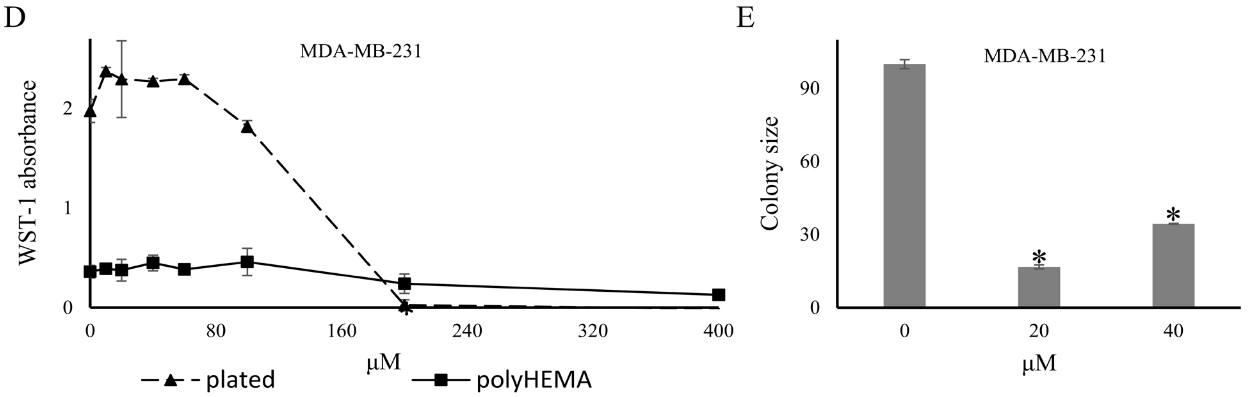

Figure S3: Verapamil effect on cell proliferation and soft agar colony formation. D) Titration of verapamil to MDA-MB-231 cells on poly-HEMA (squares, solid line) and to plated cells (triangles, dashed line). **E)** Verapamil suppression of soft agar colony formation by MDA-MB-231 cells. * indicates p < 0.05.

Supplement 4: Mechanism of action

The core program of metastasis supports the survival and expansion of cells under deadherent conditions and in foreign microenvironments. Those circumstances require an upregulation of cellular ATP production, which is accomplished via peroxide signaling and increased mitochondrial activity/biogenesis (29,59). We sought to further elucidate the mechanisms of action by the component agents of the combination therapy by measuring peroxides and mitochondrial size in PANC-1 cells grown in soft agar, with and without treatment. Expectedly, DMSO and ammonium tetrathiomolybdate suppressed the peroxide levels as well as mitochondrial biogenesis, resulting in smaller colony sizes in soft agar. Pazopanib was effective in reducing biogenesis and colony size, but less so in lowering the cellular peroxides, indicating that it acts through different pathways. Even though bumetanide showed little efficacy in inhibiting colony formation by PANC-1 cells, it nevertheless exerted a partial reduction of peroxide levels and mitochondrial size. The drug combination achieved a stronger reduction of colony size than any of the component agents, and it was accompanied by a substantial reduction in peroxides and biogenesis. Noticeably, NAC alone suppressed colony formation, but had limited efficacy in suppressing peroxides or mitochondrial size. When added to the drug combination, it exerted the paradoxical effect of increasing colony size in soft agar. The long congoing debate over the benefits or harms of antioxidants in cancer prevention and treatment may be explained, in part, by the high context-dependence of antioxidant effects.

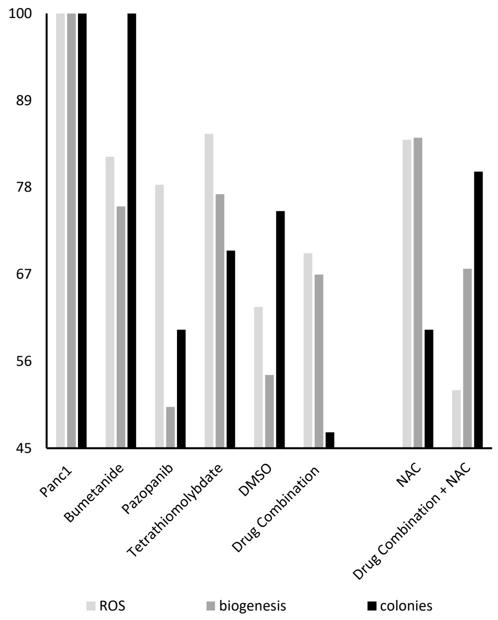

Figure S4: Mitochondrial biogenesis and reactive oxygen species under drug treatment. PANC-1 cells were grown in soft agar for 16 hours or for 5 days before measurement of intracellular peroxides with 5 µM DCFH-DA (ROS) or measurement of mitochondrial size (indicative of biogenesis) with 100 nM MitoTracker (biogenesis). The fluorescence of the untreated cells (83.51 and 72.38 relative units for peroxides, 24.20 and 31.56 relative units for mitochondrial size) was set to 100%. Every two days, 400 µl medium was added either without drugs or with pazopanib at 0.2 µg/ml, ammonium tetrathiomolybdate at 0.25 µg/ml, bumetanide at 7.5 µg/ml, DMSO at 0.13%, or NAC at 1.5 mM (all are final concentrations). Shown are the averages from 2 experiments. For comparison, the graph displays the percent inhibition of soft agar colony formation (untreated = 100%) as measured in Figures 2-6.

Following the subcutaneous injection of B16-F10 cells (shown in Figure 2E), the untreated mice and some mice from the treatment groups (no bumetanide and no marimastat) had enough tumor burden to allow the removal of a small chunk for RNASeq analysis. With 2-3 samples per group, significance of the false discovery rate (FDR) was not achievable. But the analysis trended in a confirmatory direction. According to the gene ontology resource (http://geneontology.org/), the top GO category for untreated versus no bumetanide in molecular function was GO:0070089 chloride-activated potassium channel activity (expected 0.01, fold enrichment >100, raw p-value 1.55 $x10^{-2}$) and in biological process was GO:0002007 detection of hypoxic conditions in blood (expected 0.01, fold enrichment >100, raw p-value 1.55 $x10^{-2}$). Marimastat has a broader effect spectrum through impacting the matrix microenvironment. For untreated versus no marimastat in biological process the top hits were GO:0036399 TCR signalosome assembly (expected 0.0, fold enrichment >100, raw p-value 3.49 $x10^{-3}$) and GO:0031129 inductive cell-cell signaling (expected 0.0, fold enrichment >100, raw p-value 3.49 $x10^{-3}$).

Supplement 5: Drug safety

The pharmacologic agents that make up this combination therapy have been used individually in the clinic for various conditions. By itself, each is considered safe. To ascertain that the combination of these medications does not yield adverse drug-drug interactions, we treated two non-tumor-bearing Balb/c mice (female 6 weeks) with the full treatment of pazopanib 8 mg/kg, marimastat 15 mg/kg, tetrathiomolybdate 5 mg/kg, bumetanide 2 mg/kg, DMSO 200 μl/kg every other day for 14 days. Upon completion we analyzed kidneys, livers, and lungs by hematoxylin-eosin staining in comparison to two matched but untreated mice. The mice had shown no signs of impairment at the end of the treatment period, and the organs were free from signs of toxicity (Figure S5).

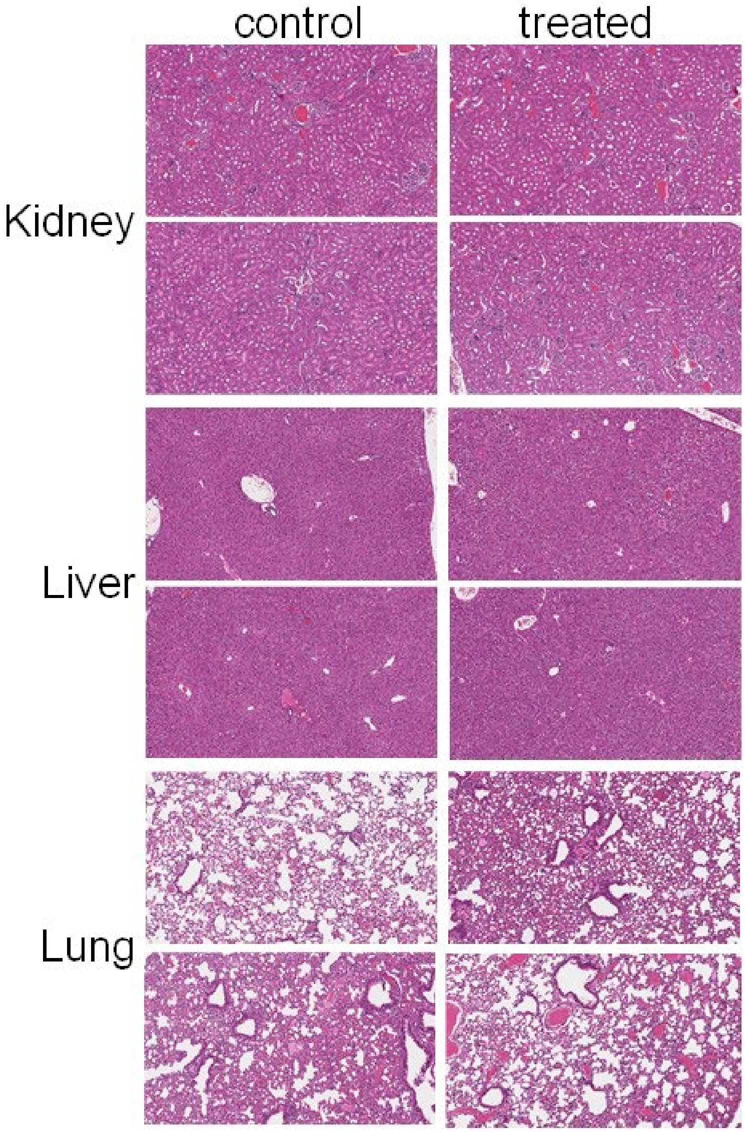

Figure S5: Drug safety. Two Balb/c mice (female 6 weeks, no tumors) were injected i.p. every other day with the anti-metastasis drug combination (pazopanib 8 mg/kg, marimastat 15 mg/kg, tetrathiomolybdate 5 mg/kg, bumetanide 2 mg/kg, DMSO 200 ml/kg). After 14 days, they were analyzed in comparison to two matched untreated mice by histology. Shown are kidneys, livers, lungs for control (left panel) and treated (right panel) mice. Each slide was stained with hematoxylin-eosin and then photographed at an original magnification of 10x.

Supplement 6: Summary of drug actions

The drugs evaluated and the model cell lines used are summarized with the testing results in Figure S6.

	MDA-MB-231		PANC-1		MIA Paca 2		ASPC1	SW1116	CCL-136
	poly-HEMA	soft agar	poly-HEMA	soft agar	poly-HEMA	soft agar	poly-HEMA	poly-HEMA	poly-HEMA
**oxidative metabolism**									
NAC	n.t.	+	n.t.	+	n.t.	–			
taurine				–					
DMSO	n.d.	+	n.d.	+	n.d.	+			
**tissue remodeling**									
pazopanib	+	+	+	+	+	+	+	+	
marimastat	n.t.	n.t.	n.t.	n.t.	n.t.	n.t.	n.t.	n.t.	
**ion homeostasis**									
bumetanide	+	+	–	–	+	+			+
tetrathiomolybdate	–	+	+	+	–	+		–	
verapamil	n.d.		n.d.		n.d.				
amlodipine	n.d.		n.d.		n.d.				

Figure S6: Individual drug effects. The display shows a summary of the monotherapy results in cell culture. Testing was done on poly-HEMA and in soft agar. + = suppression of anchorage-independence, n.d. = no differential effect, - = harmful effect (increased growth or colony formation), n.t. = not tested.

Supplement 7: Clinical information for the individual component drugs

Individual members of the drug combination, which is introduced here, have been used for various conditions or have been tested for cancer in clinical trials. The table summarizes the findings.

Figure S7: Clinical information for individual component drugs. A) Information on the individual drug components. DMSO is shown in gray because it is not FDA-approved for internal use. Marimastat and tetrathiomolybdate are displayed in light gray, because they have undergone clinical trials, but are not currently marketed for any treatment. MOA = mechanism of action. **B)** Clinical trials with monotherapies of atovaquone, bumetanide, thiomolybdate, and pazopanib have been reported.

A) basic drug data

	tissue remodeling/ vascularization		oxidative metabolism		ion dyshomeostasis	
**agent**	**pazopanib**	**marimastat**	**atovaquone**	**DMSO**	**tetrathiomolybdate**	**bumetanide**
**MOA**	angiogenesis inhibitor	MMP inhibitor	oxidative phosphorylation inhibitor	hydroxyl radical scavenger	copper chelator	NKCC channel blocker
**availability**	Votrient (Novartis)	experimental (British Biotech --> Vernalis) ?	Mepron (GSK)	dietary supplement	experimental (Pharmaceutical Care Solutions)	Bumex, Burinex, others
**current use**	renal cell carcinoma, soft tissue sarcoma		malaria, pneumonia, toxoplasmosis	interstitial cystitis (topical), iv leakage (dermal)	Wilson's disease	diuretic
**dosing**	oral tablet		oral liquid			oral or injection (i.v. or i.m.)
**dosing**	800 mg/day without food	5-75 mg 2 x daily or 10-50 mg daily	750 mg 2 x daily with food		40 mg 3 x daily + 60 mg at bedtime	0.5 - 2 mg (at 4-5 hour intervals up to 10 mg/day)
**steady state plasma concentration**	AUC 1037 h*μg/ml and Cmax 58.1 μg/mL		13.5 ± 5.1 μg/mL			
**metabolism**	CYP3A4 (major), CYP1A2 and CYP2C8 (minor)	not a substrate / inhibitor for ABCB1, CYP2C9, 2D6, 3A4, 1A2, 2C19	minimally metabolized			45% secreted unchanged; urinary / biliary metabolites by N-butyl side chain oxidation
**disposition**	mainly via feces, < 4% via urine		enterohepatic recirculation, feces >94% over 21 days; 6% in urine			45% unmodified secretion
**halflife**	31 hours		2.2–3.2 days			1-1.5 hours
**clearance**	5-6.5 days		21 days			81% in urine
**adverse effects**	Common: nausea, vomiting, diarrhea, weight loss, hypertension, trouble breathing, pain, headache, tiredness, skin dyscoloration, change in taste. Severe: hepatotoxicity, allergic reaction; tachycardia, torsades de pointes, delayed wound healing, heorrhagia, thrombemboly, gastrointestinal perforation, seizure, leukoencephalopathy		Common: nausea, vomiting, diarrhea, headache, dizzines, rash, fever, anxiety, insomnia. Severe: hepatotoxicity, hypersensitivity, hives, facial swelling, pancreatitis. Post-marketing: methemoglobinemia, thrombocytopenia, vortex keratopathy, Stevens-Johnson syndrome, renal impairmant	prolongation of bleeding time, intravascular hemolysis, vision impairment, garlic odor, Herxheimer reactions		Contraindicated: anuria. Severe: volume/electrolyte depletion, hypokalemia, ototoxicity, thrombocytopenia, allergy. Common: muscle cramps, dizziness, hypotension, headache, nausea
**potential drug interactions**	CYP3A substrates / inhibitors / inducers, grapefruit juice, antacids, simvastin, pemetrexed, lapatinib		tetracyclines, metoclopramine, co-trimoxazole, antimycobacterials, rifamycins	may facilitate the absorption of other drugs; blood thinners, sulindac		may accelerate the renal elimination of other drugs; allergy to sulfonamides, ototoxic agents, probenecid, indomethacine, lithium, antihypertensives
**sources**	drugs.com	go.drugbank.com	drugs.com	https://www.webmd.com/vi	PMID: 34426581	drugs.com
	Wikipedia		package insert	PMID: 18417375		package insert
	package insert					
**patent**	Europe 2025, expired in US	no patent active	patent expired	patents for supplement (combination)	patent active 07/2024	generic

B) Clinical trial reports

author	title	journal	URL	cancer	key findings	adverse effects
**atovaquone**						
Skwarski M, M	Mitochondrial Inhibitor Atovaquone Increases Tumor Oxygenati	Clin Cancer Res. 2021 May 1;27(9):2459-2	https://pubmed.ncbi.nlm.nih.gov/33597271/	NSCLC	reduction in hypoxic volume, reduction in hypoxia-regulated genes	none reported
Bourigault P, S	Timing of hypoxia PET/CT imaging after 18F-fluoromisonidazol	Sci Rep. 2022 Dec 16;12(1):21746.	https://pubmed.ncbi.nlm.nih.gov/36526815/	NSCLC	reduction in hypoxic volume	
**bumetanide**						
Zhou Y, Sun W	Discovery of NKCC1 as a potential therapeutic target to inhibit I	Oncotarget. 2017 Aug 12;8(39):66328-663	https://pubmed.ncbi.nlm.nih.gov/29029515/	hepatocellular carcinoma	attenuated proliferation and invasion of HCC cells in vitro, limited the HCC growth in vivo	
**thiomolybdate**						
Blockhuys S, I	Evaluation of ATOX1 as a Potential Predictive Biomarker for Te	Biomedicines. 2021 Dec 12;9(12):1887.	https://pubmed.ncbi.nlm.nih.gov/34944703/	breast cancer	ATOX1 is a predictive biomarker for TM treatment of breast cancer patients at high risk of recurrence	
Chan N, Willis	Influencing the Tumor Microenvironment: A Phase II Study of C	Clin Cancer Res. 2017 Feb 1;23(3):666-67	https://pubmed.ncbi.nlm.nih.gov/27769988/	breast cancer	improved event-free survival	common grade 3/4 toxicity was neutropenia (3.7%)
Jain S, Cohen	Tetrathiomolybdate-associated copper depletion decreases cir	Ann Oncol. 2013 Jun;24(6):1491-8.	https://pubmed.ncbi.nlm.nih.gov/23406736/	breast cancer	significant reduction in endothelial progenitor cells	grade 3/4 toxicity was neutropenia (3.1%), febrile neutropenia (0.2%), and anemia (0.2%).
Liu YL, Bager	Tetrathiomolybdate (TM)-associated copper depletion influence	NPJ Breast Cancer. 2021 Aug 23;7(1):108.	https://pubmed.ncbi.nlm.nih.gov/34426581/	breast cancer	change in TME biomarkers	
Gartner EM, C	A pilot trial of the anti-angiogenic copper lowering agent tetrathi	Invest New Drugs. 2009 Apr;27(2):159-65.	https://pubmed.ncbi.nlm.nih.gov/18712502/	colorectal cancer		TM can be safely added to IFL
Schneider BJ,	Pre-operative chemoradiation followed by post-operative adjuva	Invest New Drugs. 2013 Apr;31(2):435-42.	https://pubmed.ncbi.nlm.nih.gov/22847786/	esophageal cancer	disease-free survival and overall survival are promising	well tolerated in the adjuvant setting
Redman BG, I	Phase II trial of tetrathiomolybdate in patients with advanced kic	Clin Cancer Res. 2003 May;9(5):1666-72.	https://pubmed.ncbi.nlm.nih.gov/12738719/	kidney cancer	copper depletion, stable disease	well tolerated
Pass HI, Brew	A phase II trial of tetrathiomolybdate after surgery for malignant	Ann Thorac Surg. 2008 Aug;86(2):383-9; d	https://pubmed.ncbi.nlm.nih.gov/18640301/	malignant mesothelioma	prolonged time to progression in stage I and II patients	minimal toxicity
Brewer GJ, Di	Treatment of metastatic cancer with tetrathiomolybdate, an ant	Clin Cancer Res. 2000 Jan;6(1):1-10.	https://pubmed.ncbi.nlm.nih.gov/10656425/	metastatic cancer	effective in reducing ceruloplasmin	nontoxic
Henry NL, Dur	Phase II trial of copper depletion with tetrathiomolybdate as an	Oncology. 2006;71(3-4):168-75.	https://pubmed.ncbi.nlm.nih.gov/17641535/	prostate cancer	did not delay disease progression	1 discontinuation in 16 patients because of toxicity
Lin J, Zahurak	A non-comparative randomized phase II study of 2 doses of AT	Urol Oncol. 2013 Jul;31(5):581-8.	https://pubmed.ncbi.nlm.nih.gov/21816640/	prostate cancer	improvement in PSA kinetics	
**pazopanib**						
Narayanan S,	Phase II Study of Pazopanib and Paclitaxel in Patients With Re	Clin Genitourin Cancer. 2016 Oct;14(5):43	https://pubmed.ncbi.nlm.nih.gov/27068017/	urothelial cancer	promising overall response rate	most frequent side effects (all grades) were fatigue (63%), diarrhea (44%), nausea and vomiting (41%); hematologic toxicities (all grades) were anemia (69%), neutropenia (38%), thrombocytopenia (47%)
Taylor SK, Ch	A phase II study of pazopanib in patients with recurrent or meta	Oncologist. 2010;15(8):810-8.	https://pubmed.ncbi.nlm.nih.gov/20682606/	breast carcinoma	disease stability	grade 3-4 transaminitis, hypertension, neutropenia (14% each), grade 3 gastrointestinal hemorrhage (5%)
Lim WT, Ng C	A Phase II Study of Pazopanib in Asian Patients with Recurrent	Clin Cancer Res. 2011 Aug 15;17(16):548	https://pubmed.ncbi.nlm.nih.gov/21712450/	nasopharyngeal cacrinoma	clinical benefit rate was 54.5%	grade 3/4 toxicities included fatigue (15.2%), hand-foot syndrome (15.2%), anorexia (9.1%), diarrhea (6.1%), vomiting (6.1%)
Kim ST, Lee J	Prospective phase II trial of pazopanib plus CapeOX (capecitab	Oncotarget. 2016 Apr 26;7(17):24088-96.	https://pubmed.ncbi.nlm.nih.gov/27003363/	gastric cancer	moderate activity	grade 3 toxicities were neutropenia (15.1%), anemia (10.6%), thrombocytopenia (10.6%), anorexia (7.6%), nausea (3.0%), vomiting (3.0%)
Ahn HK, Choi	Phase II study of pazopanib monotherapy in metastatic gastroe	Br J Cancer. 2013 Sep 17;109(6):1414-9.	https://pubmed.ncbi.nlm.nih.gov/23989950/	gastroenteropancreatic neuroendocrine tumour	higher overall response rate	
Grande E, Ca	Pazopanib in pretreated advanced neuroendocrine tumors: a p	Ann Oncol. 2015 Sep;26(9):1987-1993.	https://pubmed.ncbi.nlm.nih.gov/26063633/	neuroendocrine tumor	improved progression-free survival, clinical benefit rate varied according to prior therapy	
Sgouros J, Ar	First Line Gemcitabine/Pazopanib in Locally Advanced and/or M	Anticancer Res. 2020 Feb;40(2):929-938.	https://pubmed.ncbi.nlm.nih.gov/32014937/	biliary tract carcinoma	low response rate	
Messaritakis I,	Heterogeneity of circulating tumor cells (CTCs) in patients with	Lung Cancer. 2017 Feb:104:16-23.	https://pubmed.ncbi.nlm.nih.gov/28212995/	SCLC	significant effect on different subpopulations of circulating tumor cells	VEGFR2+ CTCs as surrogate marker associated with resistance
Messaritakis I,	Dynamic changes of phenotypically different circulating tumor c	Sci Rep. 2018 Feb 2;8(1):2238.	https://pubmed.ncbi.nlm.nih.gov/29396560/	SCLC	eliminate different CTC subpopulations in patients	
Koinis F, Agel	Second-line pazopanib in patients with relapsed and refractory	Br J Cancer. 2017 Jun 27;117(1):8-14.	https://pubmed.ncbi.nlm.nih.gov/28510571/	SCLC	promising objective responses and disease control	well tolerated
Richardson D	Paclitaxel With and Without Pazopanib for Persistent or Recur	JAMA Oncol. 2018 Feb 1;4(2):196-202.	https://pubmed.ncbi.nlm.nih.gov/29242937/	ovarian cancer	not superior to paclitaxel	discontinuation for adverse events
Powles T, Sar	An indirect comparison of the toxicity of sunitinib and pazopanib	Eur J Cancer. 2012 Nov;48(17):3171-6.	https://pubmed.ncbi.nlm.nih.gov/22766517/	clear cell renal cancer		no significant difference in the overall number of toxic events (grade 1-4)
Staehler M, Pa	First-line pazopanib in intermediate- and poor-risk patients with	Int J Cancer. 2021 Feb 15;148(4):950-960.	https://pubmed.ncbi.nlm.nih.gov/32738823/	renal cell carcinoma	active	well tolerated, 4.7% hypertension
De Wolf K, Ro	Combined high dose radiation and pazopanib in metastatic ren	Radiat Oncol. 2017 Sep 22;12(1):157.	https://pubmed.ncbi.nlm.nih.gov/28938918/	renal cell carcinoma	good local control and response rates outside the radiation field	well tolerated
Sternberg CN	Pazopanib Exposure Relationship with Clinical Efficacy and Sa	Clin Cancer Res. 2018 Jul 1;24(13):3005-3	https://pubmed.ncbi.nlm.nih.gov/29330204/	renal cell carcinoma	improved disease-free survival	did not increase treatment discontinuations or grade 3/4 adverse events, with the exception of hypertension
Sweis RF, Me	Dynamic Contrast-Enhanced Magnetic Resonance Imaging as	Clin Genitourin Cancer. 2017 Apr;15(2):207	https://pubmed.ncbi.nlm.nih.gov/27634566/	metastatic renal carcinoma		
Todo M, Shiro	Usefulness of Implementing Comprehensive Pharmaceutical C	Anticancer Res. 2019 Feb;39(2):999-1004.	https://pubmed.ncbi.nlm.nih.gov/30711987/	metastatic renal carcinoma	highly effective for enhancing treatment outcomes	
Sternberg CN	An open-label extension study to evaluate safety and efficacy o	Oncology. 2014;87(6):342-50.	https://pubmed.ncbi.nlm.nih.gov/25227656/	renal cell carcinoma	response rate was 37.5%	discontinuation for disease progression (61%); adverse events were hypertension (45%), diarrhea (45%), hair color changes (44%), anorexia (30%), and nausea (25%)
Hakimi AA, Vo	Transcriptomic Profiling of the Tumor Microenvironment Revea	Cancer Discov. 2019 Apr;9(4):510-525.	https://pubmed.ncbi.nlm.nih.gov/30622105/	renal cell cancer		
Guo J, Jin J, C	Safety of pazopanib and sunitinib in treatment-naive patients wi	J Hematol Oncol. 2018 May 22;11(1):69.	https://pubmed.ncbi.nlm.nih.gov/29788981/	renal cell carcinoma		well tolerated; hypertension (grade 3, 22%) and alanine aminotransferase increased (grade 3, 12%; grade 4, 1%)
Sternberg CN	COMPARZ Post Hoc Analysis: Characterizing Pazopanib Resp	Clin Genitourin Cancer. 2019 Dec;17(6):42	https://pubmed.ncbi.nlm.nih.gov/31601514/	renal cell carcinoma		dose modifications when required because of AEs were associated with improved efficacy
Oudard S, Be	Decrease of Pro-Angiogenic Monocytes Predicts Clinical Resp	Cells. 2021 Dec 22;11(1):17.	https://pubmed.ncbi.nlm.nih.gov/35011579/	renal cell carcinoma		
Sternberg CN	A randomised, double-blind phase III study of pazopanib in patie	Eur J Cancer. 2013 Apr;49(6):1287-96.	https://pubmed.ncbi.nlm.nih.gov/23321547/	renal cell carcinoma	no significant difference in OS; post-hoc analyses suggest OS benefit	
McKay RR, Bo	Radium-223 Dichloride in Combination with Vascular Endotheli	Clin Cancer Res. 2018 Sep 1;24(17):4081-	https://pubmed.ncbi.nlm.nih.gov/29848570/	renal cell carcinoma	biologically active and safe	no dose-limiting toxicity
Chow W, Frar	Results of a prospective phase 2 study of pazopanib in patients	Cancer. 2020 Jan 1;126(1):105-111.	https://pubmed.ncbi.nlm.nih.gov/31509242/	chondrosarcoma	positive drug activity	infrequent grade 3 or higher adverse events were hypertension (26%) and elevated alanine aminotransferase (9%)
Hirbe AC, Eulo	A phase II study of pazopanib as front-line therapy in patients w	Eur J Cancer. 2020 Sep:137:1-9.	https://pubmed.ncbi.nlm.nih.gov/32712457/	soft tissue sarcoma	there is a benefit to front-line pazopanib	no new or unexpected adverse events
Kasper B, Sle	Long-term responders and survivors on pazopanib for advance	Ann Oncol. 2014 Mar;25(3):719-724.	https://pubmed.ncbi.nlm.nih.gov/24504442/	soft tissue sarcoma	long PFS and/or OS	
Kim M, Kim TI	A Phase II Trial of Pazopanib in Patients with Metastatic Alveola	Oncologist. 2019 Jan;24(1):20-e29.	https://pubmed.ncbi.nlm.nih.gov/30254189/	soft part sarcoma	modest antitumor activity	manageable toxicities
Ronellenfitsch	Preoperative therapy with pazopanib in high-risk soft tissue sar	BMJ Open. 2016 Jan 6;6(1):e009558.	https://pubmed.ncbi.nlm.nih.gov/26739732/	soft tissue sarcoma		
Urakawa H, Ka	Phase II trial of pazopanib in patients with metastatic or unrese	Cancer Sci. 2020 Sep;111(9):3303-3312.	https://pubmed.ncbi.nlm.nih.gov/32579783/	sarcoma	partial response	
Schulte B, Mo	Phase II study of pazopanib with oral topotecan in patients with	Br J Cancer. 2021 Aug;125(4):528-533.	https://pubmed.ncbi.nlm.nih.gov/34050255/	sarcoma	did not meet its primary endpoint of progression-free rate at 12 weeks	
Kollár A, Jone	Pazopanib in advanced vascular sarcomas: an EORTC Soft T	Acta Oncol. 2017 Jan;56(1):88-92.	https://pubmed.ncbi.nlm.nih.gov/27838944/	sarcoma	promising activity in hemangioendothelioma and intimal sarcoma	
Tan AR, Dowl	Phase I study of pazopanib in combination with weekly paclitax	Oncologist. 2010;15(12):1253-61.	https://pubmed.ncbi.nlm.nih.gov/21147873/	solid tumors		safe administration
Glade Bender	Phase I pharmacokinetic and pharmacodynamic study of pazo	J Clin Oncol. 2013 Aug 20;31(24):3034-43.	https://pubmed.ncbi.nlm.nih.gov/23857966/	solid tumors	evidence of antiangiogenic effect and potential clinical benefit	well tolerated in children
